# Subduction hides high-pressure sources of energy that may feed the deep subsurface biosphere

**DOI:** 10.1038/s41467-020-17342-x

**Published:** 2020-08-05

**Authors:** A. Vitale Brovarone, D. A. Sverjensky, F. Piccoli, F. Ressico, D. Giovannelli, I. Daniel

**Affiliations:** 1 Dipartimento di Scienze della Terra, Università degli Studi di Torino, Via Valperga Caluso 35, 10125 Torino, Italy; 2 Sorbonne Université, Muséum National d’Histoire Naturelle, UMR CNRS 7590, IRD, Institut de Minéralogie, de Physique des Matériaux et de Cosmochimie, IMPMC, 4 Place Jussieu, 75005 Paris, France; 3 Department of Earth & Planetary Sciences, Johns Hopkins University, Baltimore, MD 21218 USA; 4 Institute of Geological Sciences, University of Bern, Baltzerstrasse 3, CH-3012 Bern, Switzerland; 5 Department of Biology, University of Naples Federico II, Via Vicinale Cupa Cintia, 21, 80126 Napoli, Italy; 6 Institute of Marine Biological and Biotechnological Resources, National Research Council of Italy, CNR-IRBIM, Largo Fiera della Pesca, 260125 Ancona, Italy; 7 Earth-Life Science Institute, Tokyo Institute of Technology, 1 Chome-31 Ishikawacho, Ota City, Tokyo, Tokyo 145-0061 Japan; 8 Department of Marine and Coastal Science, Rutgers University, 71 Dudley Rd, New Brunswick, NJ 08901 USA; 9 Univ Lyon, Univ Lyon 1, ENSL, CNRS, LGL-TPE, F-69622, Villeurbanne, France

**Keywords:** Carbon cycle, Geochemistry, Geodynamics, Tectonics

## Abstract

Geological sources of H_2_ and abiotic CH_4_ have had a critical role in the evolution of our planet and the development of life and sustainability of the deep subsurface biosphere. Yet the origins of these sources are largely unconstrained. Hydration of mantle rocks, or serpentinization, is widely recognized to produce H_2_ and favour the abiotic genesis of CH_4_ in shallow settings. However, deeper sources of H_2_ and abiotic CH_4_ are missing from current models, which mainly invoke more oxidized fluids at convergent margins. Here we combine data from exhumed subduction zone high-pressure rocks and thermodynamic modelling to show that deep serpentinization (40–80 km) generates significant amounts of H_2_ and abiotic CH_4_, as well as H_2_S and NH_3_. Our results suggest that subduction, worldwide, hosts large sources of deep H_2_ and abiotic CH_4_, potentially providing energy to the overlying subsurface biosphere in the forearc regions of convergent margins.

## Abstract (web summary)

Geological sources of H_2_ and abiotic CH_4_ have had a critical role in the evolution of life and sustainability of the deep subsurface biosphere, yet the origins of these sources remain largely unconstrained. Here the authors show that deep serpentinization (40–80 km) during subduction generates significant amounts of H_2_ and abiotic CH_4_, potentially providing energy to the overlying subsurface biosphere.

## Introduction

Deeply sourced H_2_ and CH_4_ in fluids are potentially important contributors of energy and carbon supporting the habitability of the largest microbial habitat on Earth, the deep subsurface biosphere^1–3^. The identification of geological processes releasing these compounds is vital in understanding natural energy, carbon cycling, and the extent and magnitude of deep life. Additionally, the identification of abiotic sources of energy sources such as H_2_ and hydrocarbons provides key information about the parameter space for the emergence of life on Earth, and on where life could exist elsewhere^4–6^. Aqueous alteration of mantle rocks, or serpentinization, is considered a key process releasing H_2_ and promoting the abiotic synthesis of CH_4_ (refs. ^7,8^). Production of H_2_-CH_4_ fluids through serpentinization has been identified at mid-ocean ridges^9–11^, on-land^12^, and in the shallow forearc of subduction zones^13,14^. Strong evidence exists that deep microbial communities take advantage of serpentinite-sourced H_2_-CH_4_^15,16^, and the identification of key building blocks of life in serpentinized ultramafic rocks supports the hypothesis that these settings could have witnessed the emergence of life on Earth^4^.

Geochemical data from forearc mud volcanos and hydrothermal seeps suggest that life exists as deep as 15 km below the surface at convergent margins^14,17,18^, and that the essential carbon to sustain deep microbiological habitats in the forearc of convergent plate margins is provided by the metamorphic recycling of subducting slabs^2^. However, the composition and redox state of fluids released from subducting slabs, and therefore their capability to sustain different forms of a deep biosphere in the forearc is unconstrained^19^. Most models consider slab-derived fluids to be rather oxidized and dominated by CO_2_ (refs. ^19,20^). However, thanks to the anomalous geothermal regimes of subduction zones which stabilize serpentine minerals to depths of ~100 km^21^; serpentinization of deep-seated mantle rocks may represent a suitable environment for the genesis and migration of deep H_2_ and abiotic CH_4_. The delivery of these gases to the subsurface biosphere from the deep Earth could dramatically change our understanding of deep carbon cycling at convergent margins and the distribution and magnitude of deep life on Earth^1,22^, and potentially other planetary bodies.

Serpentinization of mantle sections at convergent margins has been identified by geophysical investigations and numerical simulations^23,24^. Furthermore, H_2_ and CH_4_-rich fluids have been detected in subduction-zone metamorphic rocks^25–27^. However, it has been proposed that serpentinization of deep-seated mantle rocks at high temperature in subduction zones may not involve Fe oxidation and formation of Fe^3+^-bearing minerals such as magnetite, thereby inhibiting the release of H_2_ and genesis of abiotic CH_4_^28^. Whether serpentinization to produce H_2_-CH_4_-rich fluids occurs at high pressure (*P*) and temperature (*T*) conditions remains an open question.

Here, we investigate the patterns of serpentinization of ultramafic rocks within the stability field of antigorite, the high-temperature serpentine polysome characteristic of high-pressure metamorphic conditions at depths >40 km in subduction zones^21,29^. We present results from three different sections of Alpine paleo-subduction complexes. We show that intense high-pressure serpentinization happened in the subducting slab and was accompanied by reducing conditions and release of H_2_-CH_4_-rich fluids at depths of about 40–80 km. As serpentinization also affects the mantle wedge above subducting slabs^24,30^, our results suggest that subduction zones may represent large source regions of H_2_ and abiotic CH_4_ on Earth, with important consequences for the mobility of deep C and the genesis of high-pressure sources of energy. In addition to H_2_ and CH_4_, our data show that other strongly reduced compounds such as H_2_S and NH_3_ can form in deep serpentinization fluids, a result that has implications for the diversity and distribution of deep subsurface communities at convergent margins.

## Results

### Relative timing of serpentinization

Constraining the timing of serpentinization in metamorphic serpentinites from mountain belts is challenging owing to the similarities between serpentinites resulting from multiple events (e.g., ocean floor; subduction; exhumation) or from single hydration events^31^. The presence or absence of specific serpentine polysomes can provide constraints on the temperature range of serpentinization. For example, lizardite is generally stable at low temperatures and antigorite at high temperatures^29,32^. But this is useful only under the assumption of single hydration events, which can hardly be expected in a large fraction of exhumed metamorphic serpentinites. Furthermore, the mineralogy and microstructures of antigorite serpentinites formed from fresh peridotites at high-pressure conditions, and of subducted oceanic serpentinites recrystallized in the antigorite stability field could be very similar. And minerals formed during seafloor serpentinization, such as magnetite, may be preserved as relict phases in metamorphosed serpentinites^33^. Consequently, recognition of the potential of deep serpentinization to generate H_2_-CH_4_-rich fluids in subduction zones is difficult.

In order to overcome this difficulty, we investigated variably serpentinized pseudotachylyte-bearing peridotites from the blueschist- to-eclogite-facies terranes of Alpine Corsica and the Italian Western Alps. Previous studies have demonstrated that the formation of pseudotachylytes, i.e., seismically produced molten rocks, in these rocks happened at high-pressure conditions during the Alpine subduction zone^34–36^. Because the formation of pseudotachylytes is inhibited in ultramafic rocks exceeding about 5 vol% serpentinization^37^, the antigorite serpentinization of the selected ultramafic pseudotachylytes and their host rocks must have happened at high pressure in the subduction zone.

**Fig. 1:**
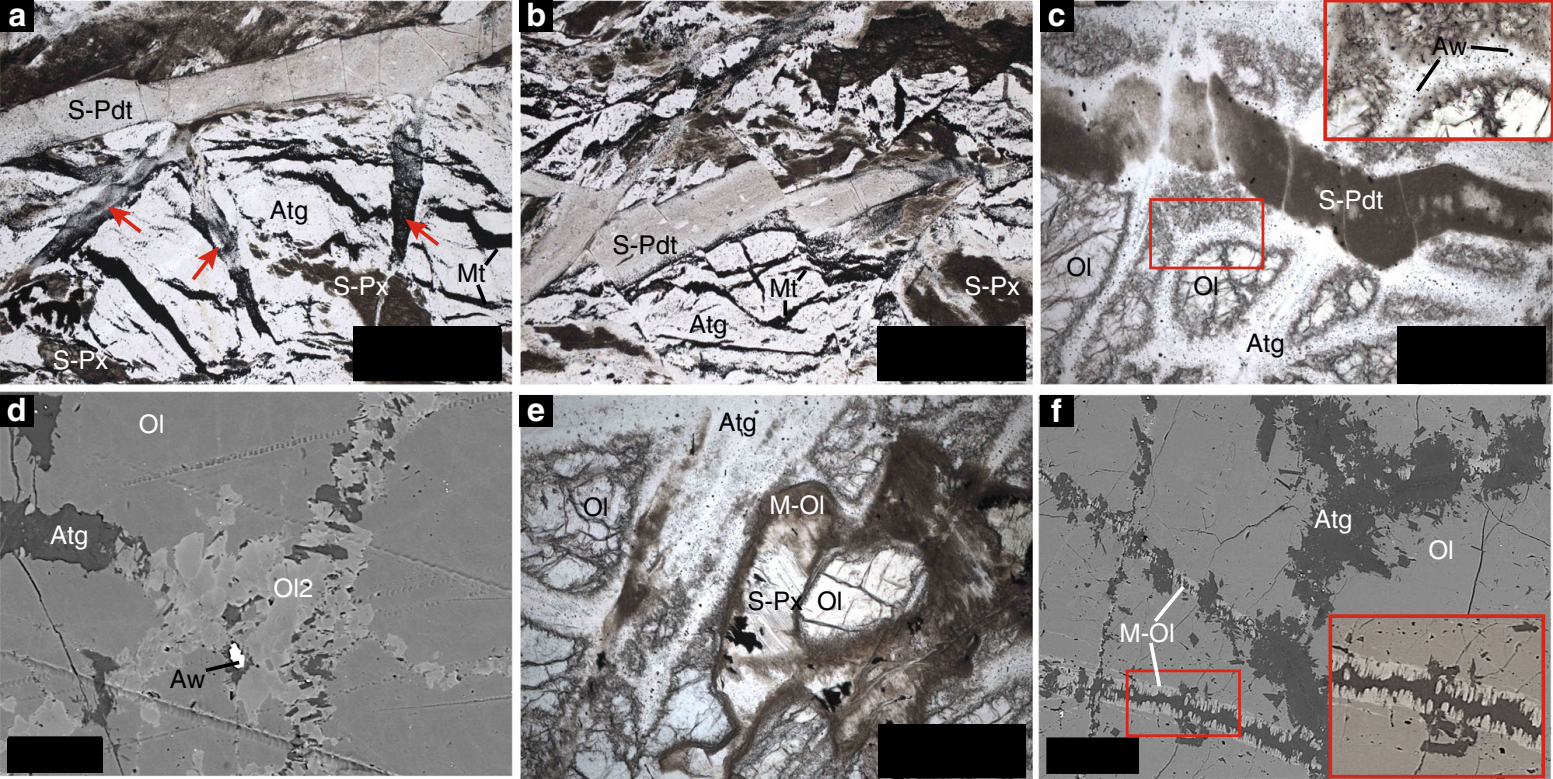
Microstructural features of antigorite serpentinization. **a**–**c** Examples of statically serpentinized pseudotachylyte (S-Pdt)-bearing peridotite from the blueschist-facies Cima di Gratera unit (**a**) and the eclogite-facies San Petrone unit (**b**, **c**). Note the absence of post-pseudotachylyte ductile deformation. **d** Backscattered-electron image showing the stage of incipient serpentinization proceeding along pre-existing deformation bands characterized by olivine subgains (Ol2) (see Supplementary Fig. 2 for details). Note the presence of awaruite (Aw) in association with antigorite. **e** Photomicrograph showing the early transformation of orthopyroxene relative to mantle olivine (see Supplementary Fig. 7 for mineralogical details). **f** Backscattered-electron image showing the formation of metamorphic olivine (M-Ol) together with antigorite during high-pressure serpentinization. Note the difference between the secondary olivine formed along a pre-serpentinization cataclastic band (**d**) and the metamorphic olivine related to the serpentinization event. Scale bars: **a**, **b** 600 µm; **c**, **e** 350 µm; **d** 20 µm; **f** 40 µm. Other mineral abbreviations: Atg antigorite, Mt magnetite, S-Px serpentinized mantle pyroxene, Ol mantle olivine.

We studied serpentinization of peridotite-hosted pseudotachylytes from the blueschist-facies Cima di Gratera, Alpine Corsica^34,36^ (metamorphic climax 430 °C and 1–1.5 GPa^38^), the eclogite-facies Lanzo massif, Italian Western Alps^35^ (2–2.5 GPa and 550–620 °C^39,40^), and Monte San Petrone, Alpine Corsica (480–530 °C and 2.2–2.4 GPa^38^). The ultramafic pseudotachylytes from the latter are reported herein for the first time (Supplementary Note 1, Supplementary Fig. 1). In these three case studies, pseudotachylyte-bearing fresh peridotite bodies are enclosed in strongly serpentinized rocks interpreted as metamorphosed oceanic serpentinites^38,40^. The latter consist mainly of antigorite + magnetite + brucite ± metamorphic olivine^36,39^. Here we assume that their formation during the pre-subduction serpentinization event (i) did not affect the studied fresh peridotite bodies, as indicated by the formation of pseudotachylytes^35,37^, and (ii) was followed by a second stage of serpentinization overprinting the pseudotachylyte-bearing peridotites at high-pressure conditions (Fig. 1).

### Patterns and conditions of high-pressure serpentinization

**Fig. 2:**
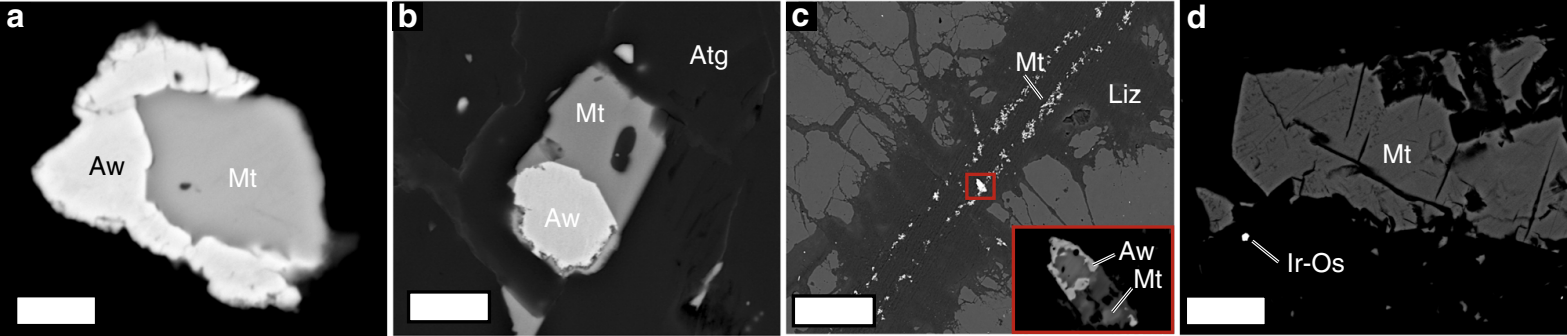
Redox buffers in the antigorite serpentinized ultramafic pseudotachylytes. **a**, **b** Awaruite and magnetite relationships in the antigorite serpentinized pseudotachylyte-bearing peridotites. **c** Magnetite and awaruite in late lizardite (Liz) veins showing the protracted reducing conditions during late-stage alteration. **d** Ir–Os particle in association with antigorite and magnetite (Supplementary Fig. 10 for details). Scale bars: **a**, **b** 2 µm; **c** 50 µm; **d** 6 µm. Other mineral abbreviations as in Fig. 1.

In the three selected case studies, the peridotite is composed of primary mantle olivine (Fo_91_), clinopyroxene, orthopyroxene and spinel, with local plagioclase veinlets, and is crosscut by deformation bands and pseudotachylytes ranging in thickness from less than 1 mm to several cm (Supplementary Note 2, Supplementary Fig. 2). The pseudotachylyte assemblage consists of the same mineralogy as the host rock, except for minor mineral compositional variations (Supplementary Table 1). The presence of spinel and absence of plagioclase in the pseudotachylytes in the three case studies constrain the formation of the pseudotachylytes to *P* > 1.1 GPa^41^, corresponding to depths greater than ~40 km. The rocks show various degrees of post-pseudotachylyte serpentinization, from incipient to rather complete (Fig. 1a–c). In order to minimize potential biases in our interpretations, we selected samples characterized by static serpentinization and no post-pseudotachylyte ductile deformation (Fig. 1a–c; Supplementary Figs. 3–5). The serpentinization proceeds along veins, grain boundaries, and along pre-existing deformation bands related to the pseudotachylyte formation event (Fig. 1d; Supplementary Figs. 3–7). Systematic cross-cutting relationships indicate that the serpentinization event post-dates the pseudotachylyte formation, as revealed by the occurrence of serpentine veins and pervasive serpentinization replacing both the pseudotachylytes and the host peridotite (Fig. 1c). Microstructural relationships enabled constraints to be placed on the high-pressure serpentinization paths, with spinel and pyroxenes being replaced first, followed by olivine (Fig. 1e; Supplementary Fig. 7). Spinel is replaced by magnetite and chlorite in all samples, including those showing little degrees of serpentinization (Supplementary Figs. 7, 9 and 10). Pyroxenes appear partially to fully replaced by diopside + brucite + antigorite + magnetite ± metamorphic olivine (Supplementary Fig. 7). Metamorphic olivine (Fo_84_) was also found together with antigorite in cross-fibre veins in samples from San Petrone (Fig. 1f). In Lanzo, metamorphic olivine and Ti-clinohumite overgrew fully serpentinized pseudotachylytes in association with antigorite and magnetite (Supplementary Fig. 8). Primary olivine was replaced by antigorite, magnetite, ubiquitous awaruite (Fe–Ni alloy), as well as various types of Fe and Ni sulfides (Fig. 2). Some samples are extremely rich in awaruite. In partially serpentinized samples, awaruite replaces magnetite, whereas in more intensively serpentinized samples magnetite rims around awaruite are found. Awaruite was also found in late lizardite veins replacing relict olivine. Tiny Ir–Os alloys were identified in samples from Monte San Petrone (Fig. 2d).

Microstructural features in partially serpentinized samples, such as delicate antigorite growth on primary olivine and antigorite cross-fibre veins cross-cutting the rock (Fig. 1c–f), indicate that the main serpentinization event happened in the antigorite stability field, i.e., at *T* ≳ 370–400 °C and depths ≳ 40 km (1 GPa) according to the estimated pressure–temperature paths in the Alpine belt^29^. This indicates that the serpentinization of the pseudotachylyte-bearing peridotites happened near peak metamorphic conditions in the blueschist-facies Cima di Gratera units (*T* climax ~430 °C/1–1.5 GPa, ~40–60 km depth), between 370 and 400 °C/1 GPa (isograd of antigorite-dominated serpentinites^29^; ~40 km depth) in the eclogite-facies Lanzo units, and 530–620 °C/2–2.5 GPa (metamorphic peak; ~80–90 km depth) in the Monte San Petrone units^40,42^.

### Redox and fluid chemistry of high-pressure serpentinization

The presence of awaruite in the studied samples indicates *f*O_2_ conditions lower than the quartz–magnetite–fayalite (QFM) buffer^43,44^. Overgrowth of awaruite on early magnetite suggests that the *f*O_2_ decreased during high-pressure serpentinization. Then magnetite growth on awaruite also suggests successive increase in *f*O_2_ during the same serpentinization event^7^. These redox patterns during progressive serpentinization are similar to those recorded by low-temperature, low-pressure serpentinization^45^ and support the potential for high-temperature, high-pressure serpentinization to generate reducing fluids.

**Fig. 3:**
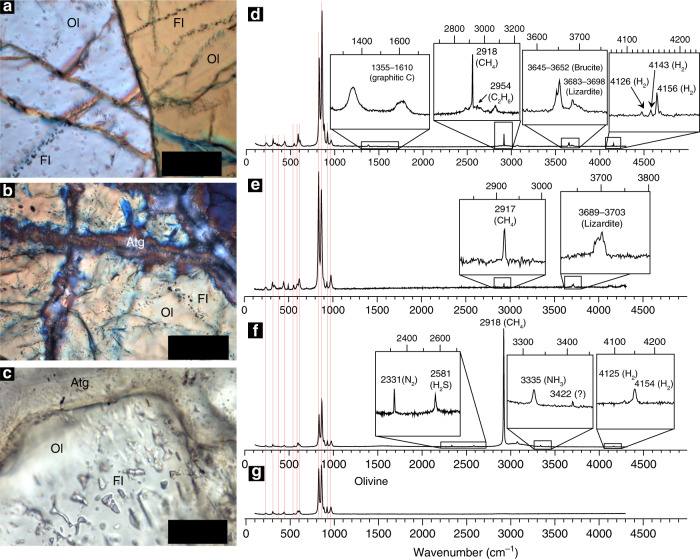
H_2_-CH_4_-rich fluid inclusions. **a**–**c** Photomicrographs of olivine-hosted fluid inclusions (FI) in partially serpentinized pseudotachylyte-bearing peridotites. Note the presence of the fluid inclusions along secondary trains propagating from serpentinized cracks and cross-cutting the primary olivine (**a**, **b**), suggesting their formation during the serpentinization events. **d**–**g** MicroRaman spectra of the fluid inclusions shown in **a**–**c**. The spectrum of the host olivine (**g**) is shown for reference. Note the presence of CH_4_ and H_2_ also in fluid inclusions free of daughter minerals, suggesting that the initial fluid trapped in the inclusions was already reduced. See Supplementary Fig. 13 for additional details. Scale bars: **a**, **b** 50 µm; **c** 20 µm.

The compositions of fluid inclusions in the rocks provide additional constraints on the reducing conditions during the high-pressure serpentinization event. Relict olivine commonly contains fluid inclusion trails that propagate from antigorite-filled fractures into the olivine (Fig. 3), constraining their formation to the high-pressure serpentinization event. MicroRaman spectroscopy of fluid inclusions reveals the presence of species characteristic of very low *f*O_2_ such as ubiquitous methane (CH_4_; 2917 cm^−1^) and H_2_ (4156 cm^−1^) (Fig. 3d–g). Ethane (C_2_H_6_), hydrogen sulfide (H_2_S), dinitrogen (N_2_), ammonia (NH_3_), and possibly another N–H compound (3422 cm^−1^) were detected in some fluid inclusions, yet not ubiquitously (Fig. 3d–f, Supplementary Note 4).

Neither H_2_O nor CO_2_ was detected in the fluid inclusions. Nevertheless, the initial presence of H_2_O in the fluid inclusions is indicated in some samples by the occurrence of hydrous step-daughter minerals inside the inclusions such as lizardite and brucite (Fig. 3d–g). The absence of a water-vapour Raman signal in fluid inclusions free of hydrous step-daughter minerals suggests either that thin films of H_2_O are present in some fluid inclusions, but are not detectable by MicroRaman spectroscopy^46^, or that syn-entrapment of immiscible H_2_O-free and H_2_O-fluids occurred. The latter hypothesis has been observed in natural samples and experimentally at pressure–temperature conditions consistent with our case studies^26,47,48^. The abundance of CH_4_ in olivine-hosted fluid inclusions indicates that carbon was present in the serpentinizing fluid and was converted to CH_4_ during the serpentinization event. Graphitic C was sporadically detected in some fluid inclusions in samples from Lanzo (Fig. 3d), and most likely reflects post-entrapment re-speciation inside the fluid inclusions.

The microstructural association of antigorite and mineral and fluid species classically attributed to low-*f*O_2_ conditions, such as awaruite, H_2_ and CH_4_, indicates that reducing conditions and genesis of H_2_ and CH_4_ were achieved during the high-pressure serpentinization event at temperatures ≳400 °C. The presence of CH_4_ and H_2_ in fluid inclusions free of post-entrapment serpentinization (Fig. 3f) suggests that these species were present in the fluid during the entrapment. The non-systematic occurrence of step-daughter minerals inside the fluid inclusions also excludes the possibility that the reduced fluid species were generated through serpentinization inside the fluid inclusions, as proposed in samples from low-temperature environments^49^. Nevertheless, the presence of step-daughter minerals potentially bearing ferric iron such as lizardite suggests that post-entrapment serpentinization and additional H_2_ and CH_4_ production may have occurred at lower temperatures inside the inclusions during exhumation. The latter hypothesis is supported by the occurrence in the host rock of lizardite veins containing low *f*O_2_ minerals such as awaruite (Fig. 2c).

**Fig. 4:**
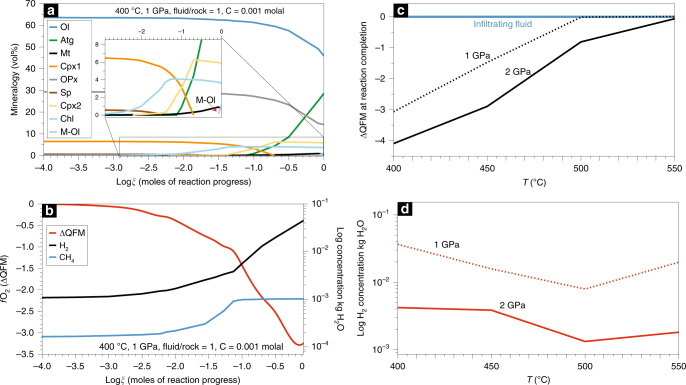
Thermodynamic modelling of high-pressure serpentinization of harzburgite and related redox evolution and H_2_ concentrations. **a** Mineralogical evolution during high-pressure serpentinization at 400 °C and 1 GPa. **b** Evolution of the *f*O_2_ and the H_2_ and CH_4_ concentrations in the fluid as a function of the reaction progress for the model presented in **a**. **c** Oxygen fugacity (as ∆Log relative to the QFM buffer —∆QFM—) for the fluid in equilibrium with either a pre-existing serpentinite at QFM (infiltrating fluid in the models, e.g., from a subducted oceanic serpentinite) and a serpentinite formed through high-pressure serpentinization at different *P* and *T*. **d** H_2_ concentration at reaction completion for different *P* and *T* conditions. The *x*-axis represents the log of the reaction progress which is equal to the number of moles of each reactant mineral destroyed per kg of H_2_O. Mineral abbreviations as in Fig. 1 except for Cpx1 mantle clinopyroxene, Opx mantle orthopyroxene, Sp mantle spinel, Cpx2 metamorphic clinopyroxene, Chl chlorite.

We performed thermodynamic calculations with the deep earth water (DEW) model^50^ in order to constrain the mineralogical, fluid, and redox patterns of high-pressure serpentinization (Fig. 4) (Methods and Supplementary Note 3). We assumed that the fluid responsible for the serpentinization of the pseudotachylyte-bearing peridotites was initially equilibrated with the surrounding serpentinite consisting of antigorite + magnetite + brucite + chlorite + olivine in the system Na–Ca–Fe–Mg–Al–Si–Cl-S–C–O–H-(±N). This fluid was then reacted with a peridotite of harzburgitic composition (olivine + clinopyroxene + orthopyroxene + spinel; Ca–Mg–Fe–Al–Si–O system) over a range of pressures, temperatures, and water-rock ratios relevant to subduction zones. The *f*O_2_ of the infiltrating fluid was buffered at QFM, with CH_4_ concentrations being 1 to 3 orders of magnitude lower than CO_2_ (Methods and Supplementary Table 2). The calculations were carried out for fluid/rock ratios ranging from 1 to 10.

Figure 4 shows the mineralogical evolution of an harzburgite interacting with a carbonate-undersaturated fluid at 400 °C and 1 GPa, i.e., approximately corresponding to the conditions of the antigorite serpentinization event in the selected blueschist-facies natural samples from the Cima di Gratera. It can be seen in Fig. 4 that the reaction proceeds by progressively transforming spinel, clinopyroxene, orthopyroxene, and olivine, consistent with the microstructural features observed in the natural samples. Magnetite starts to form during the early stages of the reaction along with chlorite, followed by diopside, and antigorite. At reaction completion, the calculations show that serpentinization reached about 50 vol% for a fluid/rock ratio equal to 1. Complete serpentinization at 400 °C and 1 GPa was obtained for fluid/rock ratios >5 (Supplementary Table 3). Metamorphic olivine is predicted to form in equilibrium with antigorite (Supplementary Table 3), as observed in the natural samples (Fig. 1e, f; Supplementary Figs. 7 and 8).

Reacting fluids equilibrated with a carbonate-bearing serpentinite result in higher degrees of serpentinization for a given fluid/rock ratio (Supplementary Table 3). This suggests that aqueous fluids equilibrated with carbon-rich rocks, e.g., metasedimentary rocks, may boost peridotite serpentinization at high-pressure conditions. This effect diminishes with increasing temperature.

At 1 GPa and 400 °C, the *f*O_2_ strongly decreases during the reaction progress as a result of magnetite formation (Fig. 4b). The highest reducing potential is observed for partially serpentinized assemblages, while calculations reaching full serpentinization show lesser decrease in *f*O_2_, as documented for low-pressure serpentinization^45^. At 1 GPa, the lowest *f*O_2_ is predicted at 400 °C (Fig. 4c). At these conditions, the H_2_ concentration in the fluid reaches about 40 mmol/kg and decreases for serpentinization degrees >50% (Supplementary Table 4).

At temperatures lower than 500 °C, the carbon-bearing fluid species are dominated by CH_4_, which is seven orders of magnitude more abundant than CO_2_. At higher temperatures (>500 °C), the degree of serpentinization decreases progressively, thus lowering the production of magnetite and H_2_. As a consequence, *f*O_2_ progressively increases towards QFM from below (Fig. 4) and higher concentrations of CO_2_ over CH_4_ are predicted (Supplementary Table 4). The CH_4_ concentration is dependent on the initial carbon content of the infiltrating fluid. For infiltrating fluids in equilibrium with a carbonate-bearing serpentinite, the CH_4_ concentrations during serpentinization reaches 19 mmol/kg at 1 GPa and 400 °C. For reference, CH_4_ concentrations in serpentinite-hosted vents along mid-ocean ridges reach 2.5 mmol/kg^9^. At 1 GPa, temperature increasing over 450 °C results in lower degrees of serpentinization for a given fluid/rock ratio, and thereby less of a decrease in *f*O_2_ and lower H_2_ and CH_4_ concentrations in the fluid (Fig. 4c). At 500 °C and 1 GPa and fluid/rock ratio = 1, the amount of magnetite coexisting with antigorite drastically decreases of about two orders of magnitude relative to temperatures <500 °C and the *f*O_2_ remains at QFM (Supplementary Table 3). Calculations at 2 GPa indicate that serpentinization of ultramafic rocks at depth consistent with eclogite-facies conditions is still effective at ~500 °C and forms about one order of magnitude more magnetite relative to the same temperature at 1 GPa. With increasing pressure, the concentration of H_2_ did not linearly follow the degree of serpentinization and the *f*O_2_ as a result of the strong decrease in solubility of gaseous H_2_ with pressure. The modelled nitrogen speciation matches the fluid inclusion data, with NH_3_ being the dominant N-species in equilibrium with a serpentinized peridotite at 1–2 GPa (Supplementary Table 4).

The calculations also show that pressure favours the formation of higher amounts of metamorphic olivine in equilibrium with antigorite and magnetite at lower temperatures (Supplementary Table 3). Based on the modelled metamorphic olivine compositions, serpentinization of the San Petrone pseudotachylytes took place at ~500 °C and 1–2 GPa, which are in agreement with the metamorphic evolution of this unit.

### Subduction-zone sources of deep H_2_ and abiotic CH_4_

Our study provides evidence that high-pressure serpentinization can take place at the expense of subducted fresh mantle rocks at blueschist-to-eclogite-facies conditions. Free water circulation in subducted mantle sections is predicted by numerical models^51^ and can promote high-pressure serpentinization. Our results show that this process is accompanied by Fe oxidation and generation of high concentrations of H_2_ and CH_4_ in the resulting fluids. We found that the *f*O_2_ of the fluids produced by high-pressure serpentinization can be up to 4 log units lower than in subducted oceanic serpentinites^52^ (Fig. 4). This provides a new and complementary view on the role of serpentinite and serpentinization on the redox state of subducting slab fluids, which have generally been considered rather oxidized^19,20,53^. Considering the potentially large volumes of peridotites being subducted annually, this process may strongly affect the redox of subduction-zone fluids and the mobility of deep C compared to more oxidized fluids^54^.

**Fig. 5:**
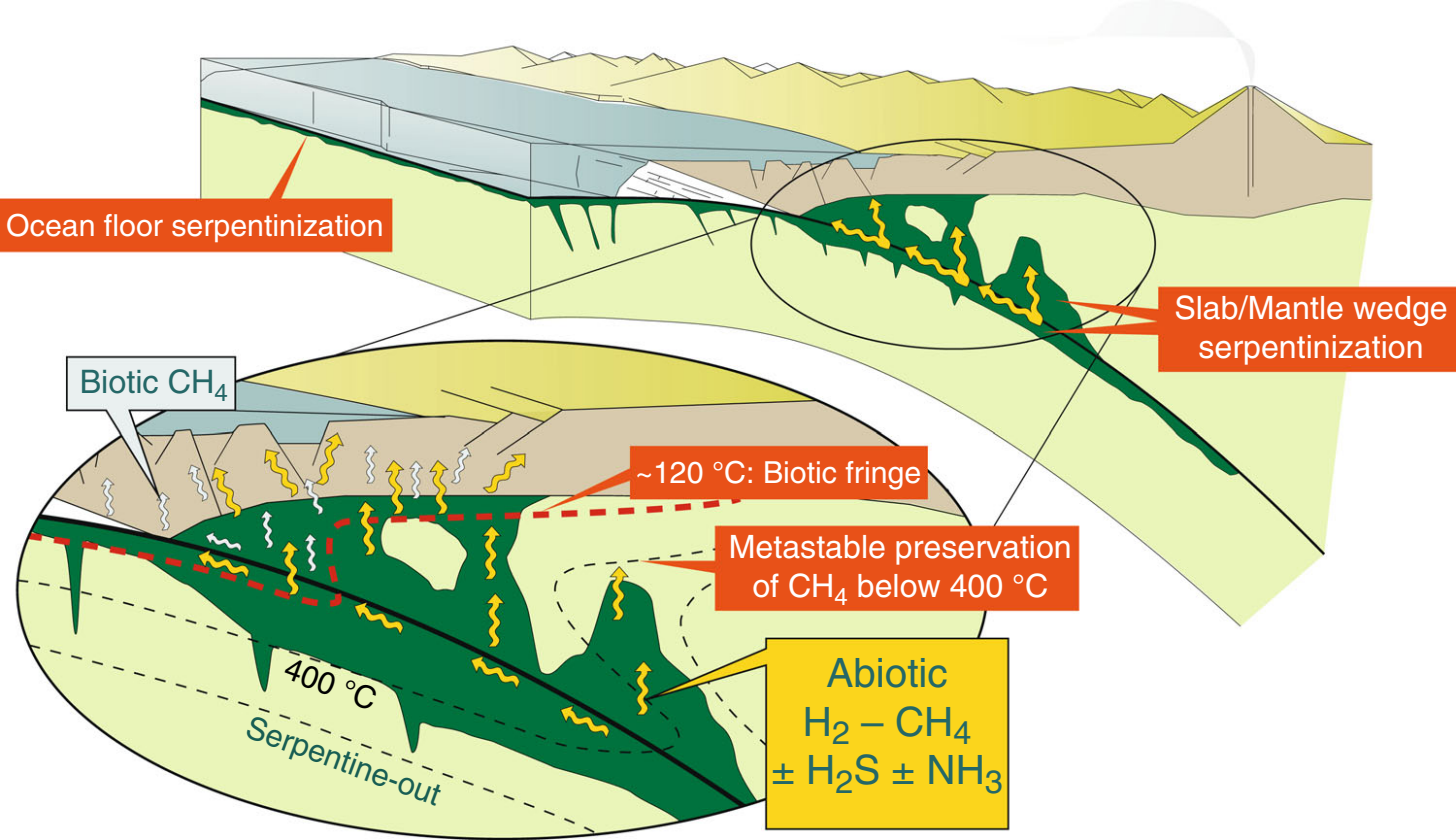
Conceptual model of high-pressure serpentinization and genesis of deep H_2_-CH_4_-rich fluids in subduction zones. Biotic release of light hydrocarbons through thermogenic processes is expected at low pressure and temperature conditions in subducted sedimentary rocks. At higher pressure and temperature conditions, production of reduced fluid species is dominantly abiogenic. High-pressure serpentinization is expected in both subducted mantle sections, where pre-existing ocean floor and slab bending serpentinization may be also present, and in in the mantle wedge. The reported 120 °C isotherm represents the current *T* limit of life, the so-called biotic fringe^77^. The 400 °C isotherm is shown to define the slab and mantle-wedge regions where the conversion of CH_4_ is kinetically inhibited (*T* < ~400 °C)^55,75,81^. The ascent of these abiotic reduced fluids may fuel deep subsurface life metabolic reactions potentially including anaerobic methane oxidation and methanogenesis. The sketch and displayed isotherms are idealized and not to scale.

At the temperatures in subduction zones that we considered, high-pressure serpentinization produces favourable conditions for overcoming the kinetic inhibition of the abiotic conversion of dissolved carbon into CH_4_, in marked contrast to shallower, lower temperature environments^55,56^. Moreover, the presence of catalysts such as Fe–Ni and platinum-group alloys, here shown to be widespread in serpentinized ultramafic rocks formed at high pressures, could greatly enhance the kinetics of CO_2_ hydrogenation to CH_4_^57^. At even greater depths in subduction zones, where serpentinites dehydrate, there is also the potential for these rocks to release reduced fluid species such as H_2_, CH_4_, and H_2_S^52^. We suggest that these strongly reduced fluids may be preserved during up-slope migration through subducted mantle sections (Fig. 5). Percolation of these fluids through more oxidized subducted oceanic crustal and sedimentary lithologies may result in conversion of CH_4_ into graphitic C or carbonate minerals into less reduced C-bearing fluid species (e.g., CO, HCOOH, CO_2_, or HCO_3_^−^), or may promote reduction of carbonate-bearing lithologies and additional CH_4_ generation^26,58,59^.

Although large uncertainties still exist regarding the definition of serpentinization patterns in the mantle wedge above subducting slabs, several examples of inferred high-pressure, mantle-wedge serpentinized peridotites exhibit mineral assemblages comparable to the samples presented in this study^60,61^. Therefore, our petrological data and thermodynamic modelling can be extrapolated to portions of the hydrated mantle wedge above subducting slabs^24,30^, where variable degrees of high-pressure serpentinization occur along more than 55,000 km of forearc mantle domains^24^. Emissions of H_2_ and CH_4_ have been identified in the Izu-Bonin-Mariana subduction and ascribed to the serpentinization of the shallow forearc mantle to depths potentially reaching 27 km^13^. Our study suggests that the source areas and magnitudes of H_2_-CH_4_-rich fluids at convergent margins may be much deeper and much larger, respectively, than previously expected.

Our data enable provisional estimates of high-pressure H_2_ and abiotic CH_4_ release from worldwide subduction zones. Considering extents of serpentinization ranging from ~10 to ~45%, consistent with predictions of mantle-wedge serpentinization worldwide^24^ (Supplementary Note 3), the highest and lowest H_2_ production through antigorite serpentinization are expected at 1 GPa (~35 km depth) and 400 °C (~0.3 kg H_2_ per m^3^ of rock; based on the instantaneous H_2_ concentration at reaction completion), and 2 GPa (~70 km depth) and 500 °C (9 × 10^−3^ per m^3^ of rock), respectively (Fig. 4d; Supplementary Table 4). Using these instantaneous concentrations as conservative values for the H_2_ production during serpentinization, it is estimated that the minimal annual production of H_2_ from high-pressure serpentinization of the mantle wedge lies between 9 × 10^−3^ and 0.3 Mt (Supplementary Note 3). Variations in the reacting peridotite and fluid compositions may result in additional heterogeneity. The associated release of CH_4_ can greatly vary as a function of parameters such as the concentration of C in the serpentinizing metamorphic fluids, the kinetics of the hydrogenation reaction, and the presence of catalysts. For example, from ~35 to 70 km depth (1–2 GPa) and temperature of 400–550 °C and infiltrating fluid ranging from largely carbonate-undersaturated to carbonate-saturated, the annual CH_4_ production through partial serpentinization of the mantle wedge could be in the range of 2.3 × 10^−3^ to 1.0 Mt (Supplementary Note 3). These numbers would be greatly increased by considering the cumulative H_2_ production instead of an instantaneous concentration, higher C concentrations, and additional fluxes from lizardite serpentinization in the mantle wedge (<35 km depth), from slab-serpentinization and from post-entrapment serpentinization of aqueous fluid inclusions^49^ in high-pressure ultramafic rocks, and from serpentinite dehydration^52^.

Global estimates of geological abiotic H_2_ and CH_4_ production vary widely. Serpentinization at mid-ocean ridges is a major source of these gases in the Earth’s lithosphere^11,12^, with H_2_ and CH_4_ production reaching ~0.02–1.4 and ~1.1–1.9 Mt/y^62^, respectively. Global estimates for shallow forearc serpentinization and obducted ophiolites are not available to date^12^. High-pressure serpentinization in subduction zones is currently not included in the global inventory of geological abiotic H_2_ and CH_4_ sources^12,63^. However, our results and preliminary estimates on high-pressure serpentinization are consistent with—and may potentially exceed—the range of H_2_ and CH_4_ production proposed for mid-ocean ridges. We therefore propose that deep serpentinization of slab and mantle-wedge ultramafic rocks may represent the largest source of natural energy in the form of H_2_ and abiotic CH_4_ in Earth’s lithosphere. In warm subduction zones, mantle-wedge serpentinization and the resulting integrated H_2_ and CH_4_ fluxes are maximized but limited to shallower depths compared to cold subduction zones. High integrated H_2_-CH_4_ fluxes from the mantle wedge are expected for thermal gradients characteristic of Neoarchean to Cambrian subduction^64^ (Supplementary Note 4), even though the possibility for deep serpentinization reactions to have occurred in early subduction systems is not an established feature. Nevertheless, similar warm geothermal gradients are predicted in active convergent margins such as Cascadia^23,24^ (Supplementary Fig. 12). In cold subduction zones characteristic of the Phanerozoic, high-pressure serpentinization may have extended to greater depths compared with warm subduction zones but is predicted to be less pervasive at least in the mantle wedge^24^. However, because the concentration of H_2_ and CH_4_ is higher in fluids in equilibrium with partially serpentinized rocks compared to strongly serpentinized ones^4^, it is expected that mantle-wedge domains in cold subduction zones bear the greatest potential to produce deep, highly concentrated H_2_-CH_4_ fluids.

### Nitrogen and ammonia in deep serpentinization fluids

Nitrogen enters subduction zones mainly as organic nitrogen present in sedimentary organic matter and ammonium ions (NH_4_^+^) bound in phyllosilicates^65^. Prograde dehydration of these minerals is expected to release nitrogen, with N_2_ being considered the dominant dissolved N-bearing species in subduction fluids^65,66^. In this study, nitrogen-bearing species were identified in fluid inclusions from several samples (Fig. 3f, Supplementary Fig. 13). Subducting slabs, especially subducted sedimentary rocks, host orders of magnitude more nitrogen than mantle rocks^67^. Nitrogen species have not been documented in CH_4_-bearing fluids inclusions in ultramafic rocks from mid-ocean ridges, shallow forearc settings, and ophiolites^49^. The involvement of fluids released by dehydration of subducted sediments in the process of high-pressure serpentinization, however, may explain the nitrogen-rich fingerprints observed in our samples relative to shallower settings. The presence of abundant nitrogen in fluid inclusions may therefore help to differentiate between CH_4_-bearing fluids generated by serpentinization at mid-ocean ridges and shallow settings relative to the equivalent gases produced through high-pressure serpentinization. Additionally, the abundance of nitrogen in fluid inclusions may help to identify the source of serpentinizing fluids at high-pressure conditions.

The identification of NH_3_ in the studied fluid inclusions provides new perspectives on the chemistry of high-pressure metamorphic fluids in subduction zones. Ammonia in geological fluid is mainly produced through biological and thermal degradation of organic matter. The formation of NH_3_ in the rocks we studied, however, was likely related to the strongly reducing conditions achieved abiotically during high-pressure serpentinization that stabilized NH_3_ over N_2_ in the fluid, as predicted by the thermodynamic modelling results (Supplementary Table 4). To the best of our knowledge, our study represents the first documentation of NH_3_ in metamorphic fluids at concentration levels detectable by MicroRaman spectroscopy^68^. The genesis, preservation, and evolution of NH_3_ in metamorphic fluids may provide new insights on the cycle of nitrogen in subduction zones. Ammonia can be a powerful ligand for transition metals in geologic fluids^69^. The presence of platinum-group elements particles as well as abundant alloys in the studied samples (Fig. 2d) suggests that deep serpentinization reactions and genesis of NH_3_ may represent a potential pathway for the transport of transition metals in subduction zones. The re-speciation of NH_3_ into nitrogen species with weaker ligand properties can therefore control the deposition of transition metals at convergent margins. Thermal decomposition of NH_3_ to N_2_ in the presence of catalysts may also represent an additional source of natural H_2_ (2NH_3_ → N_2_ + 3H_2_)^69^. Oxidation of NH_3_ can lead to the formation of N_2_ and H_2_O^69^. The conversion of NH_3_ to N_2_ also represents an important step in the evolution of nitrogen isotopic signatures of deep geological fluids^70^.

The circulation and transformation of deep NH_3_-bearing fluids may also play an important role in sustaining the deep subsurface biosphere at shallower depths (see next section). Last, the identification of NH_3_ in fluids generated by the alteration of ultramafic rocks at high-pressure, low-temperature conditions may provide insights on the evolution of other planetary interiors. As an example, accreted NH_3_ is proposed to have played an important role in the evolution of Titan’s atmosphere^71^. Interestingly, Titan’s core pressure and temperature conditions may overlap with the conditions investigated in mantle rocks in this study^72^, suggesting that cold subduction zones on Earth may provide a possible new terrestrial analogue for planetary science studies.

## Discussion

Reduced fluid species such as CH_4_, H_2_, H_2_S, and NH_3_ are key compounds in planetary evolution, prebiotic chemistry, and metabolism^4,73,74^. The release of such reduced fluid species from subducting slabs is generally considered to happen at relatively low temperature and pressure conditions through biotic/thermogenic process related to the recycling of subducted biogenic substances^75,76^ (Fig. 5). At the higher pressure–temperature conditions characteristic of subduction-zone metamorphism, more oxidized conditions are traditionally expected^20^. The results presented in this study, however, show that fluids highly concentrated in such compounds can be formed abiotically down to 40–80 km depth through high-pressure serpentinization, potentially in high amounts, and along thousands of km at convergent plate boundaries (Fig. 5). These processes occur way outside the parameter space for deep life^77^. However, the migration of these fluids towards shallower depths within the limits of the deep subsurface biosphere may have important implications for the sustainment of deep microbial communities at convergent margins (Fig. 5). While a growing body of literature has highlighted the global role of the subsurface biosphere in contributing to the deep carbon cycle^1,78^, relatively few studies have so far investigated the deep subsurface communities in subduction zones^3,79^, and to what extent this deep life depends on deeper carbon recycling at convergent margins^2^.

Although the possibility that reducing high-pressure fluids could migrate and eventually reach the deep subsurface biosphere remains poorly constrained, and could be the subject of future studies, the geological conditions at which this migration could occur strongly support this hypothesis. The hydrating mantle wedge would in fact represent an ideal means to maintain the reduced state of these fluids during upward migration^53^ before crustal recycling. Relative to CO_2_-rich fluids, which are expected to promote carbonate precipitation in mantle-wedge peridotites^20,80^ and at shallower depths into the crust^2^, reduced CH_4_-rich fluids should not promote the same reactions. Possible exceptions are additional serpentinization reactions—and thereby additional reducing potential—and graphite precipitation. Cooling of these metamorphic fluids makes CH_4_ thermodynamically more stable relative to CO_2_^75^. This suggests that the preservation of CH_4_ formed through high-pressure serpentinization is enhanced during upslope migration in the forearc (Fig. 5). Moreover, when the fluids reach temperatures less than about 400 °C, conditions that represent large portions of forearc mantle wedges, CH_4_ may be kinetically inhibited from reaction as has been suggested for decades^55,75,81^. Kinetic inhibition of CH_4_ reactivity would therefore be expected to result in a state of metastable equilibrium in the fluid in which the relative stabilities of many aqueous organic species (e.g., formate and acetate) are greatly enhanced, offering an ideal opportunity for support of an opportunistic microbial community.

Recent discoveries strongly suggest that deep subsurface microbial communities present in the forearc and arc continental crust respond to deeply sourced carbon and volatile species^2,3^. In particular, the study by Barry et al.^2^ carried out in the Costa Rica convergent margin identified significant differences in the starting composition of the carbon end-members feeding into their geobiological precipitation sink, and propose that these differences are directly related to primary differences in the carbon species released from the subducting slab. Most relevant to this discussion, the signature of slab fluids was visible throughout the forearc and into the volcanic front. This suggests pervasive migration of fluids from the subducting slab thorough the mantle wedge into the overlying forearc crust where they finally interact with the subsurface biosphere. During the fluid migration towards the pressure–temperature range suitable for life to flourish^82^, it is plausible that the reduced species identified in this study, e.g., CH_4_, H_2_, NH_3_, and H_2_S, encounter more oxidized redox couples therefore fuelling deep subsurface life metabolic reactions^13,83^. Possible oxidants include CO_2_, Fe^3+^ present in the more oxidized crustal rocks, partially oxidized organic carbon and halogenated compounds from diagenetic processing of surface-derived organic matter, as well as oxidized sulfur and nitrogen species derived from the entrainment of surface and meteoric waters into deep hydrothermal systems at convergent margins^3^. Possible microbial metabolic strategies supported by the ascent of reduced metamorphic fluids include methanogenesis, anaerobic methane oxidation potentially coupled to a variety of oxides^84^ as well as a variety of exergonic catabolic strategies. Anaerobic methane oxidation is generally coupled to sulfate reduction^84^, but it has been recently linked also to NO_3_- and MnO reduction^85,86^ and can theoretically be coupled to other electron acceptors including Fe^3+^ and zerovalent sulfur^84^. Members of microbial taxa known to carry out these reactions have been reported in several continental subsurface ecosystems^78^. Additionally, H_2_, CO and NH_3_ found to be abundant in the deep metamorphic fluids in this study might fuel widespread chemolithoautotrophy as recently reported at convergent margins^2,3^ and other subsurface continental settings^87^. Heterotrophic and fermentative members of the community commonly reported in other continental settings^78,87^ may be supported by the aqueous organic species resulting from abiotic synthesis (e.g., formate and acetate^88^). Overall, the presence of a large supply of reduced volatile species has the potential to support diverse microbial assemblages at convergent margin.

Despite the large predicted flux, the surface emissions of similar deeper fluids released by high-pressure serpentinization, however, may not be obvious, owing to the reworking by oxidative fluid–rock interactions and microbial activity within the crust. A similar overprint of geochemical and biological factors has been shown to mask potentially large carbon fluxes in the Costa Rica convergent margin^2^. Abiotic and biogenic recycling of high-pressure CH_4_ and associated reduced compounds should therefore be considered in the interpretation of forearc volatile signatures and in the inventory of deep carbon processes.

Overall, in light of recent investigations into the role of subsurface microbes at convergent margins^2,3,17^, our results suggest that high-pressure serpentinization is potentially an important source of reduced volatiles to the deep subsurface biospheres of convergent margins. Considering that low temperature and pressure serpentinization also takes place at subduction zones in the shallow forearc mantle and in obducted ophiolites, we propose that convergent margins may have represented the major source of H_2_ and abiotic CH_4_ from different depths to the surface biosphere. The identification of these different sources within the subsurface biosphere represents an important challenge for future work.

## Methods

### Sample preparation and microanalysis

Samples from the Cima di Gratera, Monte San Petrone, and Lanzo were studied by optical microscopy, scanning electron microscopy, and energy dispersive X-ray analysis (SEM-EDX), electron microprobe, and Raman spectroscopy.

For each sample, a 30-µm-thick polished thin section was prepared. For all samples containing fluid inclusions but one (OF3223), a 100-µm-thick bi-polished thin section was also prepared to avoid potential contaminations (e.g., by epoxy). The thin sections selected for electron microscopy and microprobe were carbon coated. MicroRaman analyses were conducted prior to C coating or on uncoated thick sections.

SEM-EDX analyses were performed with a Zeiss Ultra 55 field emission gun (FEG) SEM at IMPMC. The analyses were done with a working distance of 7.5 mm and operated at 15 kV with a 120 μm aperture. Backscattered-electron mode was used to investigate chemical heterogeneities using an Angle Selective Backscattered Detector (AsB) or an Energy Selective Backscattered Detector (EsB). Energy dispersive X-ray spectrometry (EDXS) maps were acquired using an EDXS QUANTAX system equipped with a silicon drift detector XFlash 4010 (Bruker). Data were processed with the software Esprit (Bruker).

Mineral chemistry was determined by wavelength-dispersive spectrometry using a JEOL JXA 8200 superprobe at the Institute of Geological Sciences (University of Bern). Analytical conditions included 15 keV accelerating voltage, 20 nA specimen current, 40 s dwell times (including 2 × 10 s of background measurements), and a beam diameter of 2 μm. Higher current and larger beam size were used for spinel measurements (50 nA and 5 μm). For silicate minerals, nine oxide compositions were measured, using synthetic and natural standards: wollastonite (SiO_2_), anorthite (Al_2_O_3_), wollastonite (CaO), almandine (FeO), olivine (MgO), tephroite (MnO), bunsenite (NiO), rutile (TiO_2_), and spinel (Cr_2_O_3_). For oxide minerals, eight oxide compositions were measured, using synthetic and natural standards: almandine (FeO), spinel (Al_2_O_3_), olivine (MgO), tephroite (MnO), bunsenite (NiO), rutile (TiO_2_), spinel (Cr_2_O_3_), and sphalerite (ZnO).

MicroRaman spectra and maps were acquired using the integrated micro/macro-Raman LABRAM HRVIS (Horiba Jobin Yvon Instruments) of the Interdepartmental Center “G. Scansetti” (Department of Earth Sciences, University of Torino, Italy). Excitation lines at 532 nm (solid-state Nd laser and 80 mW of emission power) were used with Edge filter and a grating of 600 grooves/mm. Calibration was performed using the 520.6 cm^−1^ Si band. The laser power on the sample was ~2 mW for a ×100 objective. Acquisition times were set at 20 s for three accumulations for solid phases and 160 s for five accumulations for fluid inclusions, with a laser spot of 2 μm.

### Thermodynamic modelling

High-pressure serpentinization was simulated with the DEW model^50^ and the EQ3/EQ6 software^89^ with a modified Berman thermodynamic database^90^ (see also Supplementary Note 3 for additional information and discussion). Firstly we calculated with EQ3 the composition of a fluid in equilibrium with a serpentinite assemblage consisting of antigorite + magnetite + brucite + chlorite + olivine, which best represents the general mineralogical composition of serpentinites at blueschist-to-eclogite-facies conditions^29,39^. The *f*O_2_ of the equilibrium was set at QFM (quartz–fayalite–magnetite buffer). The initial value of the fluid *f*O_2_ was chosen on the basis the predicted *f*O_2_ conditions for a fluid equilibrated with the assemblage antigorite + magnetite + olivine + chlorite + brucite by Piccoli et al.^52^. In our oceanic serpentinite samples hematite was never observed, indicating that *f*O_2_ conditions never exceeded the hematite–magnetite buffer. Nevertheless, it is worth noting that the chosen initial fluid *f*O_2_ does not affect the final *f*O_2_ condition at equilibrium after fluid–rock interaction. Choosing higher initial *f*O_2_ condition will only lead to higher ∆*f*O_2_ (Fig. 4c in the main text), thus our initial *f*O_2_ conditions give more conservative results.

The molality of carbon in the fluid was set at values between 0.001 and 0.05, which encompass the values of carbon molality for in equilibrium with carbonate-undersaturated to carbonate-saturated serpentinite at 400–500 °C and 1–2 GPa based on the EQ3 calculations. EQ6 was then used to model the interaction between the EQ3 fluid and a harzburgite assemblage consisting of olivine, orthopyroxene, clinopyroxene, and spinel. The composition of the main solid solutions in the EQ3 and EQ6 calculations were set based on the mineral compositions analysed in the samples. Fluid/rock ratios from 1 to 10 were considered.

## Supplementary information

Supplementary Information

## Data Availability

The main data generated during this study are available in Supplementary Tables 1–4. Additional information is available from the corresponding author on reasonable request.

## Code availability

The code used for thermodynamic modeling is available at http://www.dewcommunity.org.
