# Supplementary material for: Subduction hides high-pressure sources of energy that may feed the deep subsurface biosphere
Source: Nat Commun. 2020 Aug 5;11:3880. doi: 10.1038/s41467-020-17342-x (PMC7406650; doi:10.1038/s41467-020-17342-x)
Supplement: Supplementary file 1 — Supplementary Information [file 41467_2020_17342_MOESM1_ESM.pdf]

Subduction hides high-pressure sources of energy that may feed the deep subsurface biosphere

A. VITALE BROVARONE[1,2], D. A. SVERJENSKY[3], F. PICCOLI[4], F. RESSICO[1], D. GIOVANNELLI[5,6,7,8], I. DANIEL[9]

[1]Dipartimento di Scienze della Terra, Università degli Studi di Torino, Italy
[2]Sorbonne Université, Muséum National d'Histoire Naturelle, UMR CNRS 7590, IRD, Institut de Minéralogie, de Physique des Matériaux et de Cosmochimie, IMPMC, 75005 Paris, France
[3]Department of Earth & Planetary Sciences, Johns Hopkins University, Baltimore, MD 21218, USA
[4] Institute of Geological Sciences, University of Bern, CH-3012 Bern, Switzerland
[5]Department of Biology, University of Naples Federico II, Naples, Italy
[6]Institute of Marine Biological and Biotechnological Resources, National Research Council of Italy, CNR-IRBIM, Ancona, Italy
[7]Earth-Life Science Institute, Tokyo Institute of Technology, Tokyo, Japan
[8]Department of Marine and Coastal Science, Rutgers University, New Brunswick, NJ, USA
[9] Univ Lyon, Univ Lyon 1, ENSL, CNRS LGL-TPE, F-69622 Villeurbanne France

Supplementary Material

This file provides extended discussion and figures of the distribution and features of the newly discovered pseudotachylyte-bearing peridotites from the eclogite-facies San Petrone unit (**Note 1**); the microstructures and patterns of serpentinization of the three selected case studies (**Note 2**); the thermodynamic modelling results (**Note 3**); the potential implications of high-pressure serpentinization in modern and ancient subduction zones (**Note 4**); and the composition of fluid inclusions deduced by MicroRaman spectroscopy (**Note 5**).

Mineral abbreviations: Ol: primary olivine; Ol2: pseudotachylyte-related olivine; M-Ol: metamorphic olivine; Cpx: clinopyroxene; Opx: orthopyroxene; S-Cpx: serpentinized clinopyroxene; S-Opx: serpentinized orthopyroxene; Sp: spinel; Mt: magnetite; Atg: antigorite; Liz: lizardite; Brc: brucite; Di: diopside; Chl: chlorite; Aw: awaruite; Ti-Chu: Ti-clinohumite; Pdt: pseudotachylyte; S-Pdt: Serpentinized pseudotachylyte.

Supplementary Note 1

Field and petrographic features of the Monte San Petrone ultramafic pseudotachylytes

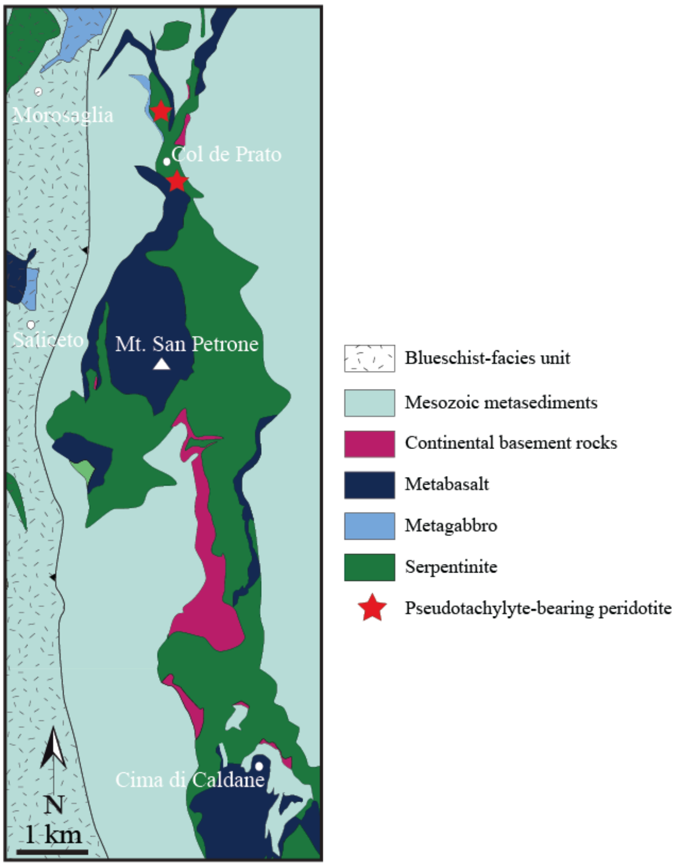

Supplementary Figure 1. Geological maps of the eclogite-facies San Petrone unit showing the distribution of the newly discovered ultramafic pseudotachylytes. Regional geological framework after Vitale Brovarone et al. [1].

The eclogite-facies Monte San Petrone unit belongs to the Schistes Lustrés complex of Alpine Corsica[2] and represents a section of subducted and metamorphosed transitional continental-to-oceanic lithosphere of the Alpine Tethys Ocean[3,4]. The unit consists of a basal body of serpentinites overlain by a variable tectonostratigraphic suite including meta-oceanic basalts, Mesozoic metasediments, and slivers of continental basement rocks[3].

Two bodies of rather fresh peridotite were discovered in the northern part of the unit (Supplementary Figure 1) and included in the basal serpentinite. Pseudotachylytes were found in both bodies (Supplementary Figure 2). A transitional zone separates the fresh peridotite from

the enclosing serpentinite and is characterized by the presence of mostly statically serpentinized pseudotachylyte-bearing peridotites (Fig. 2c-d).

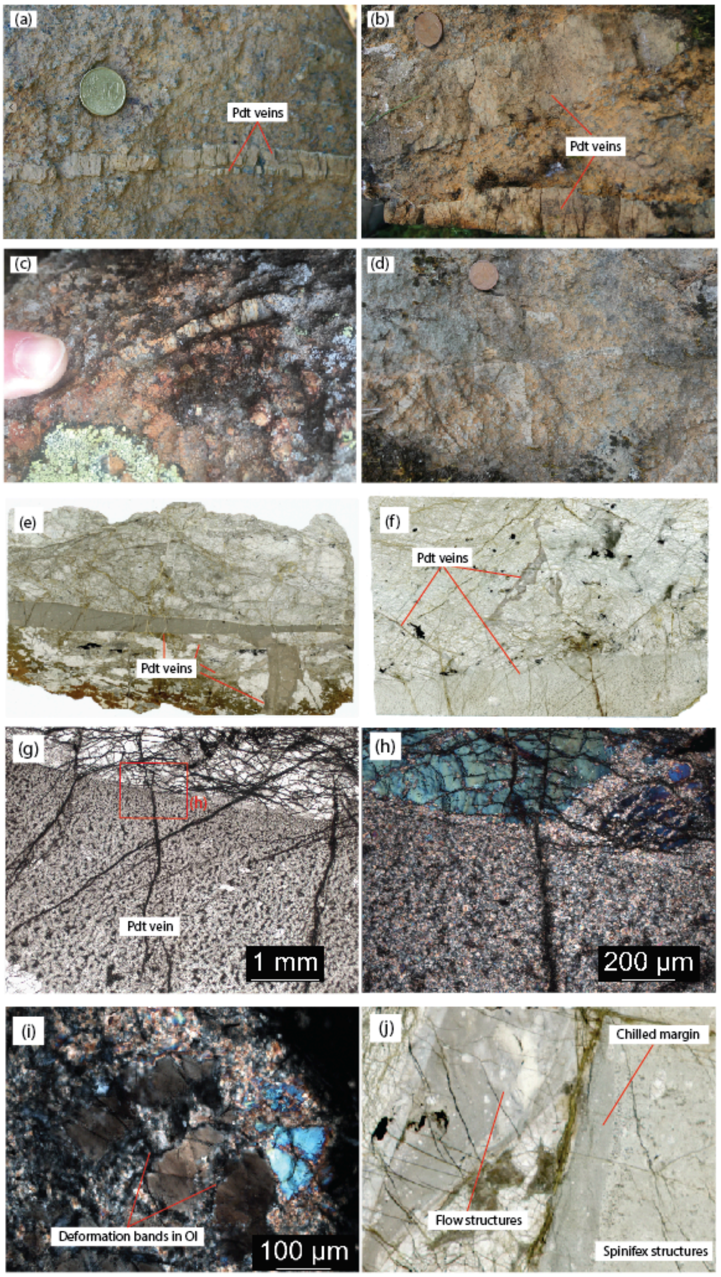

Supplementary Figure 2. a,d) Field appearance of pseudotachylyte-bearing peridotites in the eclogite-facies San Petrone unit. On the weathered surface, the serpentinized pseudotachylyte veins show a lighter colour with respect to the enclosing serpentinized peridotite. **e,f)** Scans of thin sections (about 4 cm in length) of fresh pseudotachylyte-bearing peridotites. Note the presence of multiple pseudotachylyte generations, and the different microstructural patterns as a function of the vein thickness. **g,h)** Characteristic microstructures of the Monte San Petrone ultramafic pseudotachylytes. Note the spinifex structure indicating a very fast quenching of the pseudotachylyte melt.

The San Petrone pseudotachylytes share the same general macroscopic and microscopic features as the subduction zone Gratera and Lanzo ultramafic pseudotachylytes[5-10]. The peridotite ranges from pristine to intensely shuttered and brittlely deformed. The pseudotachylyte veins are generally sharp and commonly show injection veins on one side of the main frictional plane. From the field to the microscale, at least two successive and cross-cutting generations of pseudotachylyte veins were identified. The thickness of both vein generations is variable and ranges from few mm to several cm. The largest pseudotachylytes from the San Petrone unit reach ~10-15 cm in thickness.

At the microscale, the mantle assemblage exhibits intense shuttering and grain-size reduction along deformation bands (Supplementary Figure 2e-h), as documented by previous studies on the Gratera and Lanzo ultramafic pseudotachylytes[5,7,10,11]. This deformation increases towards the pseudotachylyte veins. Deformation bands related to the pseudotachylyte event and catting through olivine crystals are also characterized by a strong grain size reduction with formation of olivine subgrains (Supplementary Figure 2i). Backscattered electron microscope imaging along these deformation bands reveals the presence of zoned olivine subgrains (Ol2), with cores showing Mg# equivalent to the primary olivine (Mg# = 0.91), and Fe-richer rims (Mg# = 0.85-0.87). These rims are interpreted to have formed either during the pseudotachylyte event, or during the successive serpentinization event. Microstructural investigations indicate that the serpentinization event started along these deformation bands (Fig.1).

The microstructures of the pseudotachylyte veins generally vary with the vein thickness. Small veins are characterized by a dark-brown matrix including variably deformed clasts of the host peridotite and commonly display flow structures (Supplementary Figure 2j). Large veins exhibit spinifex structures with individual rods up to ~1cm long and decreasing in size towards the vein selvages (Supplementary Figure 2g-h). The fresh pseudotachylyte veins are

characterized by fine-grained granoblastic aggregates of the same mineralogy observed in the host peridotite, including olivine, ortho- and clinopyroxene, and spinel. Spinel forms tiny crystals dispersed in the granoblastic aggregates of the pseudotachylyte, including within individual granoblastic spinifex rods. This feature indicates that the spinel found within the pseudotachylyte veins formed during the quenching of the vein in the subduction zone.

Selected representative analyses of the main minerals identified in the San Petrone rocks are reported in Table S1, whereas for the other two case studies, data can be found in the literature[9-11].

	COR18-47												COR18-39a2	
	**Ol1**	**Ol1**	**Ol2**	**Ol2**	**M-Ol**	**M-Ol**	**Opx**	**Cpx**	**Atg**	**Atg**	**Sp**	**Sp-Pdt**	**Atg**	**Atg**
**SiO_2**	40.57	40.85	39.82	40.05	39.92	40.39	55.15	51.75	43.31	42.91	—	—	42.80	42.81
**TiO_2**	0.00	0.00	0.00	0.01	0.03	0.02	0.04	0.09	0.00	0.00	0.04	0.13	0.05	0.02
**Al_2O_3**	0.01	0.03	0.00	0.24	0.08	0.08	3.25	4.10	0.22	0.56	35.51	40.11	4.56	3.32
**Cr_2O_3**	0.01	0.00	0.02	0.02	0.00	0.00	0.79	1.50	0.00	0.05	31.74	23.11	0.17	0.25
**FeO**	9.05	8.87	14.59	12.39	14.42	14.05	5.41	2.07	3.06	3.05	15.84	16.90	2.65	2.72
**MnO**	0.14	0.13	0.35	0.31	0.36	0.33	0.14	0.10	0.02	0.04	0.36	0.24	0.02	0.01
**MgO**	51.17	50.52	44.68	46.32	45.02	45.95	32.70	15.74	40.06	39.56	15.17	17.56	36.68	37.41
**CaO**	0.05	0.04	0.06	0.02	0.01	0.01	2.24	23.87	0.02	0.02	—	—	0.07	0.06
**NiO**	0.40	0.43	0.00	0.01	0.05	0.04	0.09	0.08	0.02	0.00	0.18	0.26	0.25	0.20
**Na_2O**	—	—	—	—	—	—	0.03	0.69	—	—	—	—	—	—
**ZnO**	—	—	—	—	—	—	—	—	—	—	0.37	0.45	—	—
**Total**	101.40	100.87	99.58	99.37	99.88	100.87	99.84	99.99	86.71	86.18	99.22	98.75	87.25	86.80
**atom unit**														
**Si**	0.97	0.98	0.97	0.98	0.99	1.00	1.91	1.89	2.00	1.99	—	—	1.98	1.98
**Ti**	0.00	0.00	0.00	0.00	0.00	0.00	0.00	0.00	0.00	0.00	0.00	0.00	0.00	0.00
**Al**	0.00	0.00	0.00	0.01	0.00	0.00	0.13	0.18	0.01	0.03	1.21	1.33	0.25	0.15
**Cr**	0.00	0.00	0.00	0.00	0.00	0.00	0.02	0.04	0.00	0.00	0.73	0.51	0.01	0.01
**Fe^{2+}**	0.18	0.18	0.30	0.25	0.30	0.29	0.03	0.06	0.12	0.12	0.32	0.25	0.10	0.13
**Fe^{3+}**	—	—	—	—	—	—	0.13	0.00	—	—	0.06	0.15	—	—
**Mn**	0.00	0.00	0.01	0.01	0.01	0.01	0.00	0.00	0.00	0.00	0.01	0.01	0.00	0.00
**Mg**	1.83	1.81	1.63	1.69	1.66	1.70	1.69	0.86	2.76	2.73	0.66	0.74	2.53	1.73
**Ca**	0.00	0.00	0.00	0.00	0.00	0.00	0.08	0.94	0.00	0.00	—	—	0.00	0.00
**Ni**	0.01	0.01	0.00	0.00	0.00	0.00	0.00	0.00	0.00	0.00	0.00	0.01	0.01	0.01
**Na**	—	—	—	—	—	—	0.00	0.05	—	—	—	—	—	—
**Zn**	—	—	—	—	—	—	—	—	—	—	0.01	0.01		
**Total**	3.0	3.0	2.9	2.9	3.0	3.0	4.0	4.0	4.9	4.9	3.0	3.0	4.9	4.0
**Mg#**	**0.91**	**0.91**	**0.85**	**0.87**	**0.85**	**0.85**								

Supplementary Table 1: Compositional analyses of the main minerals in the Monte San Petrone rocks.

Supplementary Note 2

Field and microstructural features of serpentinized pseudotachylyte-bearing peridotites

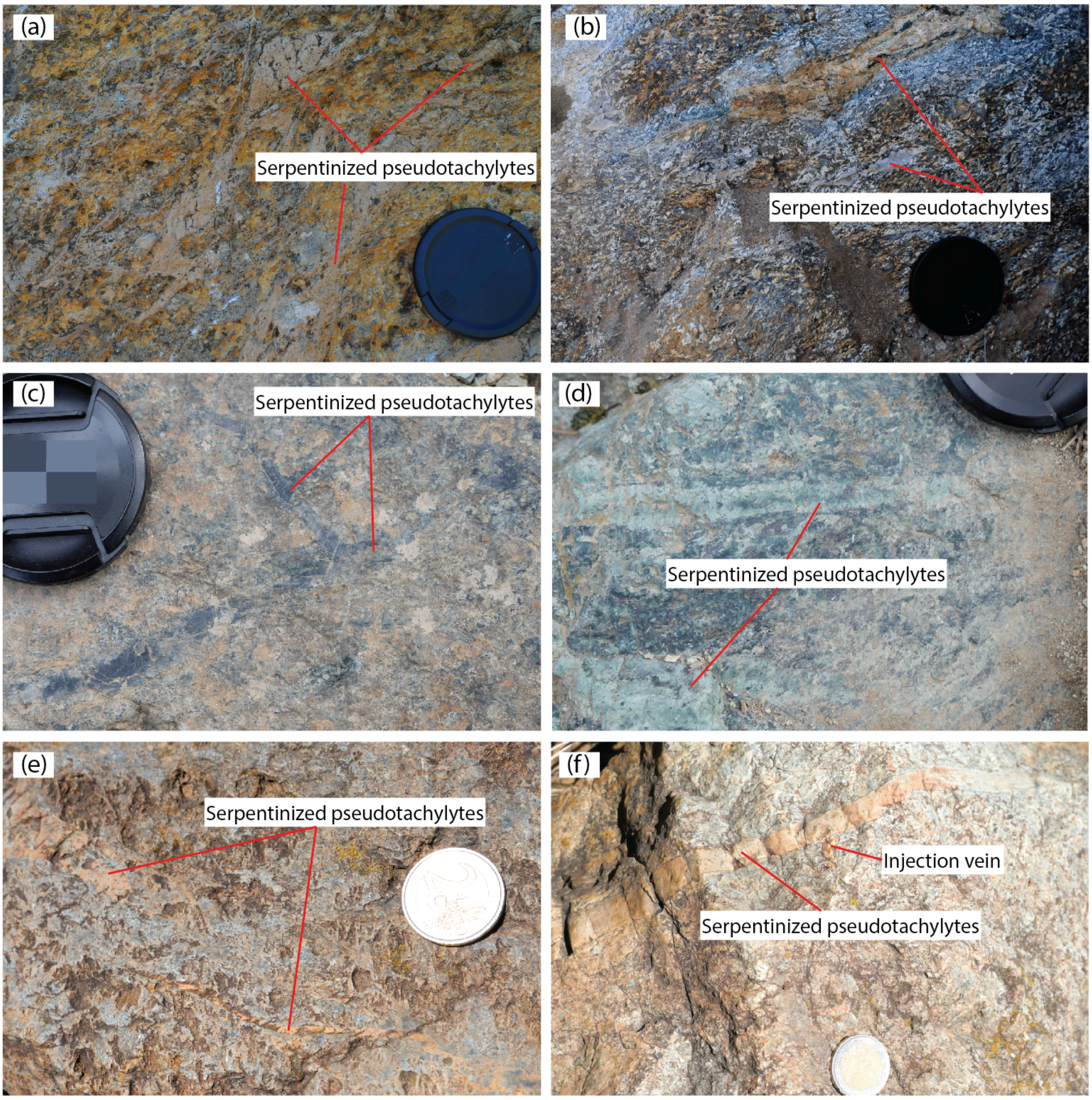

Supplementary Figure 3. Examples of statically serpentinized pseudotachylytes from the Cima di Grater (**a-b**), Monte San Petrone (**c-d**), and Lanzo (**e-f**) unit. Note the structural preservation of the pseudotachylyte vein, now partially to completely replaced by antigorite serpentinite.

Variably serpentinized pseudotachylyte-bearing peridotites were collected from the three selected case studies. In the three cases, samples were collected from transitional zones separating the fresh, pseudotachylyte-bearing peridotite from the surrounding pseudotachylyte-

free serpentinite. This transitional zones contain heavily serpentinized pseudotachylyte-bearing peridotites showing static serpentinization features and preservation of the pseudotachylyte structures (Supplementary Figure 3).

For each study area, samples characterized by different degrees of serpentinization were considered. Serpentinization initiates along small cracks, grain boundaries, and along pre-existing deformation bands related to the pseudotachylyte-formation event (Fig. 1). In several samples showing very little serpentinization, only lizardite was detected by Raman spectroscopy. Because the formation of lizardite may happen at relatively low-temperature and pressure conditions, in this work, only samples containing antigorite were considered.

In the least serpentinized samples, antigorite + magnetite ± awaruite form along fracture networks defining the typical kernell structure (Supplementary Figure 4). Lizardite was also found in these samples and, besides late veins cross-cutting the antigorite fabrics, they appear to be mainly related to Al-bearing precursors such as pyroxenes, which could extend the stability field of lizardite to higher T conditions[12].

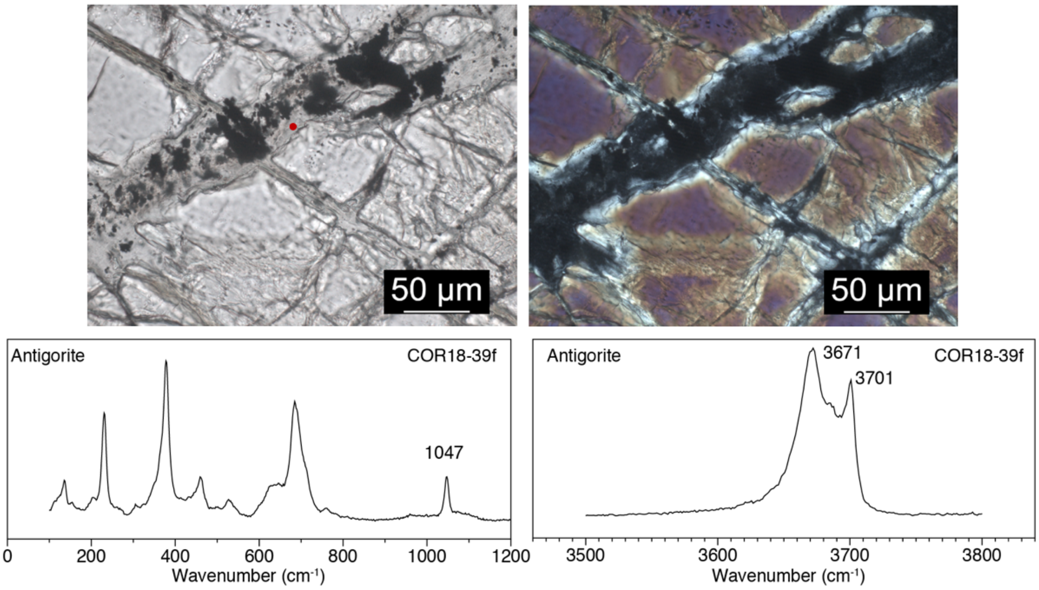

Supplementary Figure 4. Sample COR18-39f, eclogite-facies San Petrone unit. The photomicrographs show the weak serpentinization of this sample mainly developing along microcracks. The red dot refers to the Raman spot analysis, which shows features characteristic of antigorite (e.g. sharp band at 1047 cm^{-1} and O-H stretching at 3671-3701 cm^{-1}).

The more pervasively serpentinized samples contain dominant antigorite (Supplementary Figure 5-6), with lizardite limited to structurally late veins. In several completely serpentinized samples no lizardite was found. The microstructural analysis indicates that the aqueous alteration firstly affected the mantle pyroxenes and spinel, with formation of complex coronitic reactions (Supplementary Figure 7). Mantle pyroxenes are completely replaced by diopside, antigorite, magnetite, brucite and metamorphic olivine (Supplementary Figure 7a-d). Spinel is partially replaced by magnetite and chlorite (Supplementary Figure 7e-f).

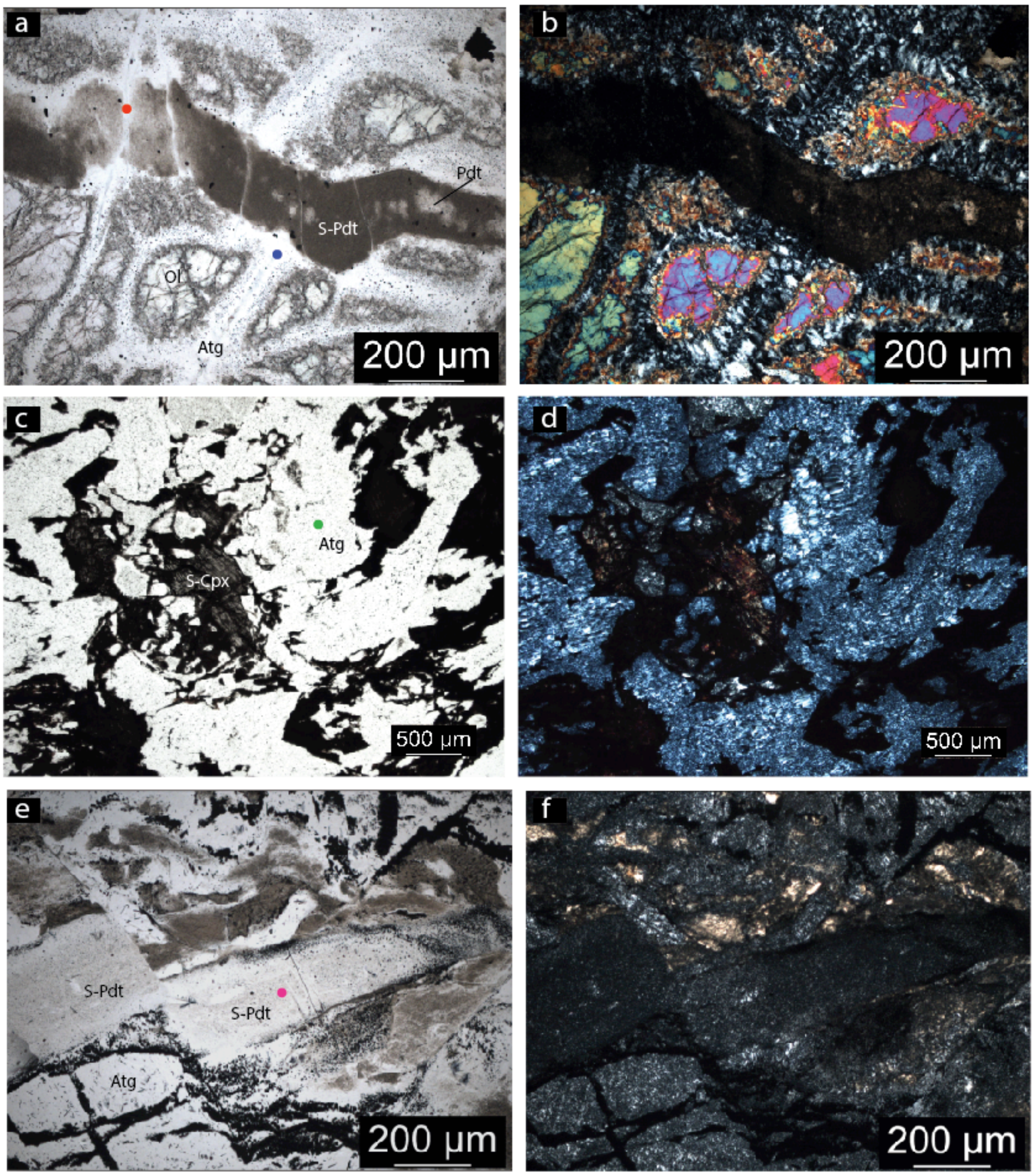

Supplementary Figure 5. Microphotographs showing the intense serpentinization of the rock and the enclosed pseudotachylyte vein. The coloured dots refer to the Raman spot analyses reported Supplementary Figure 6.

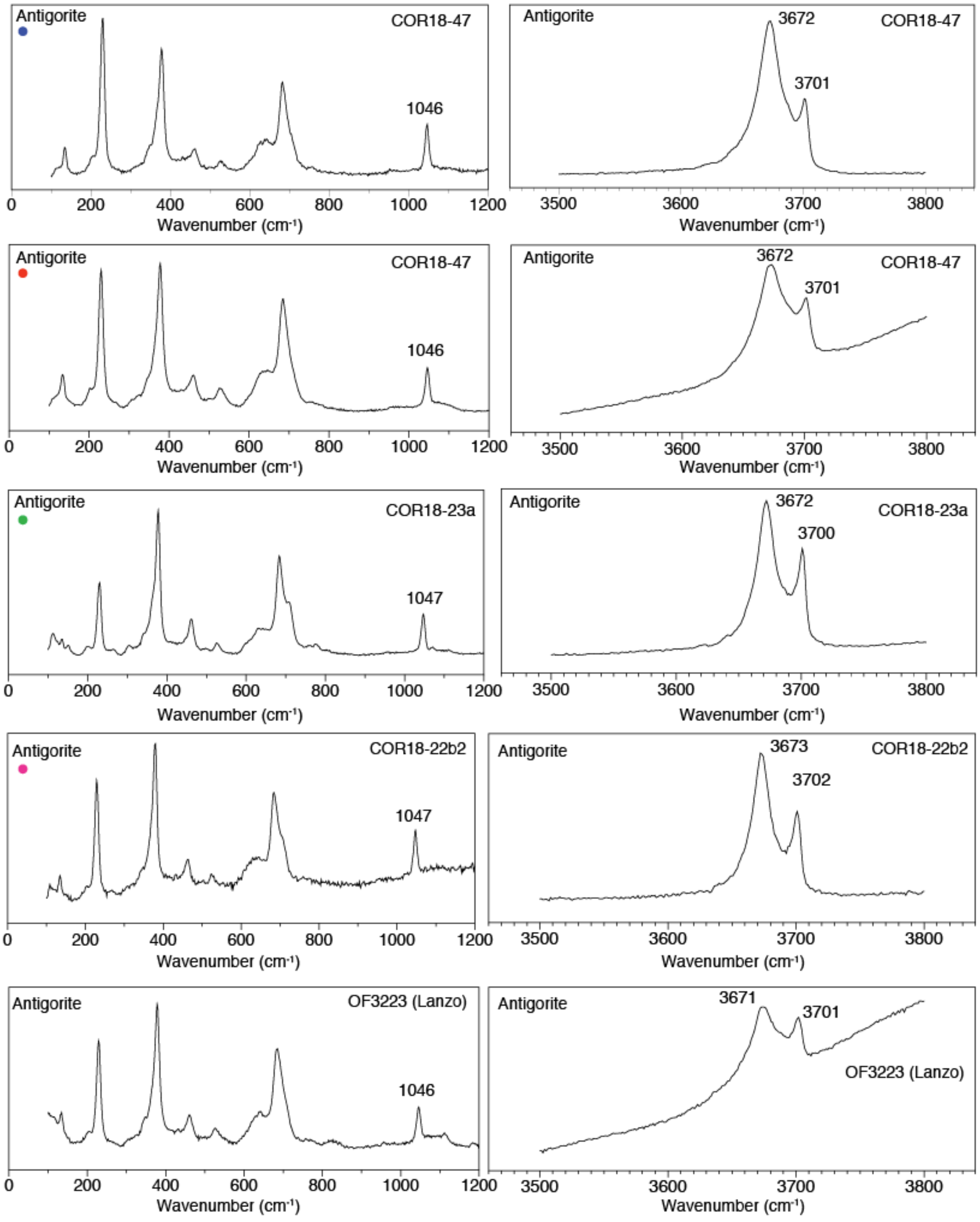

Supplementary Figure 6. a-b) Raman spectra of serpentine in the serpentinized pseudotachylyte-bearing peridotites. The coloured dots refer to the spot analyses of Supplementary Figure 5.

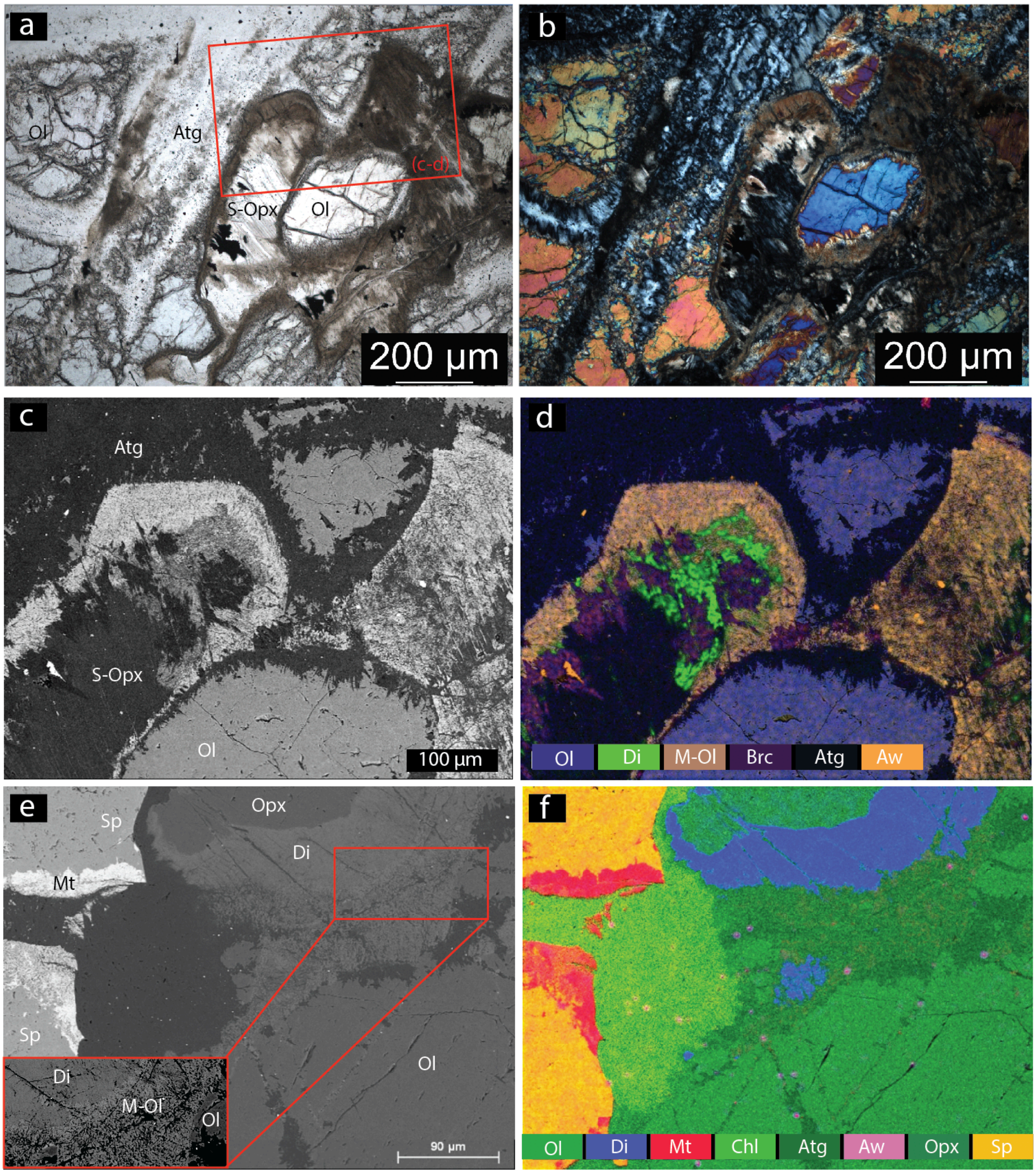

Supplementary Figure 7. a-b) Microphotographs of an intensely serpentinized pseudotachylyte-bearing peridotite. Note the more intense alteration of pyroxene relative to olivine. **c,d)** Scanning Electron Microscope (SEM) backscattered-electron and Energy Dispersive X-ray (EDX)-based mineralogical map of olivine and mantle pyroxene alteration. Note the formation of a corona of metamorphic olivine around the altered pyroxene, and the formation of diopside, brucite and antigorite in the core. **e-f)** SEM backscattered and X-ray-based mineralogical map of olivine and spinel alteration. Awaruite formed during antigorite serpentinization (see Fig. 1a in the main text for microstructural details of the analysed particle). Mineralogical maps in (d) and (f) were produced by merging SEM-EDX compositional maps via the Esprit software (Bruker).

Metamorphic olivine was found in serpentinized pseudotachylytes from Lanzo and San Petrone. In the Lanzo samples, metamorphic olivine is widespread in intensely serpentinized pseudotachylyte samples. It commonly statically grew across the boundaries separating the pseudotachylyte veins from the host peridotite, now consisting of antigorite + magnetite + metamorphic olivine + Ti-clinohumite (Supplementary Figure 8). These microstructures clearly constrain the formation of metamorphic olivine later than the pseudotachylyte formation event.

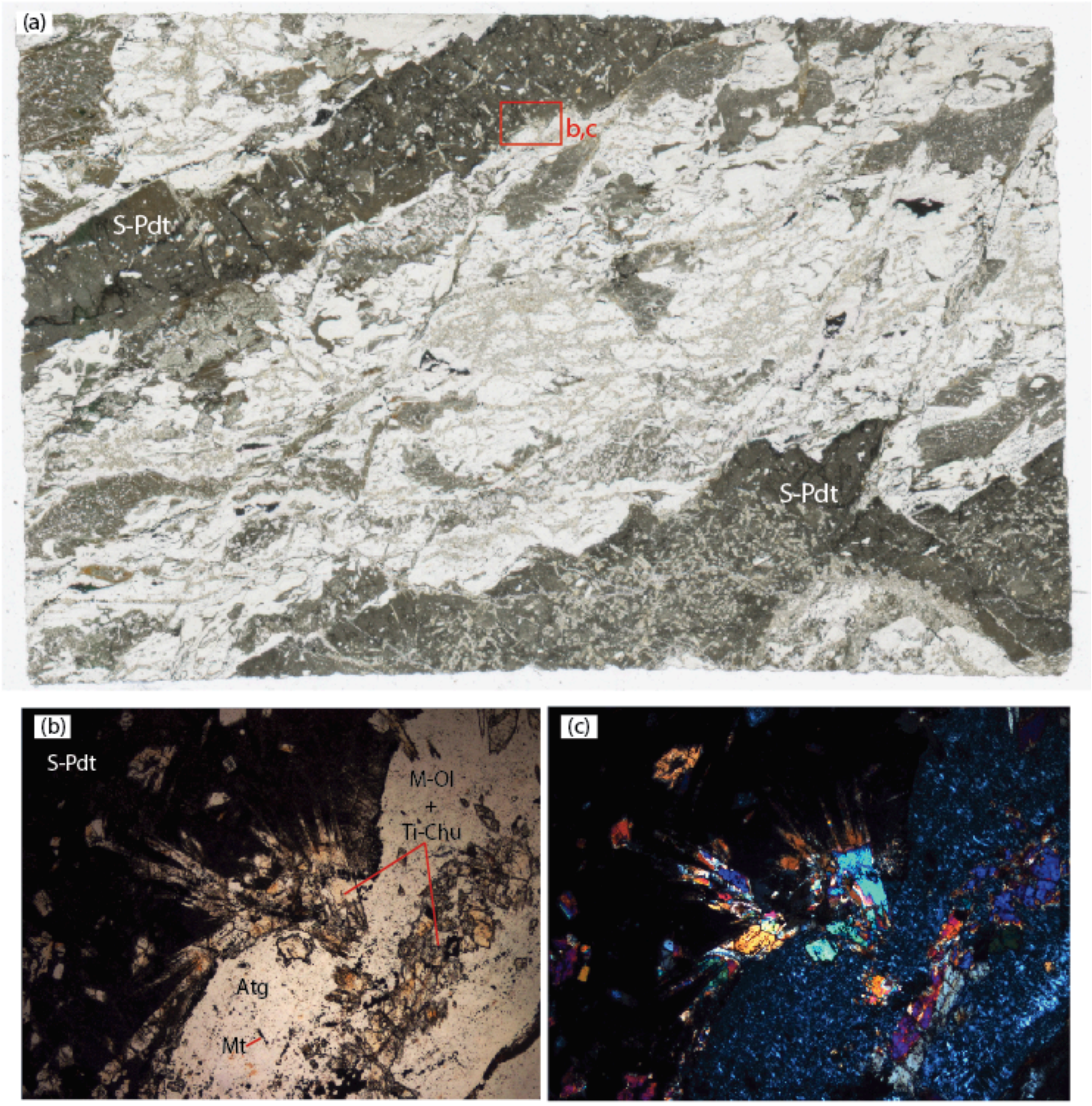

Supplementary Figure 8. a) Scan of a thin section of serpentinized pseudotachylyte-bearing peridotite from Lanzo. **b,c)** Photomicrographs showing the growth of metamorphic olivine and Ti-clinohumite (orange colour) across the boundary separating a pseudotachylyte vein from the host peridotite now mainly consisting of antigorite. b) plane-polarized light; c) cross-polarized light.

Opaque minerals comprise magnetite, sulphides (mainly pentlandite), Fe-Ni (awaruite), Ir-Os, and Wo-Co-Fe-Cr alloys, and native metals (identified by X-Ray microanalyses). Awaruite was found in all samples, including those showing rather complete serpentinization. In most cases, awaruite predates magnetite and Fe-Ni sulphides (Supplementary Figure 9), even though opposite microstructural relationships were also locally observed. Ir-Os alloys were found in one sample from the eclogite-facies Monte San Petrone unit, Alpine Corsica (Supplementary Figure 10). Tungsten alloys were found included in magnetite in one sample from the Cima di Gratera unit.

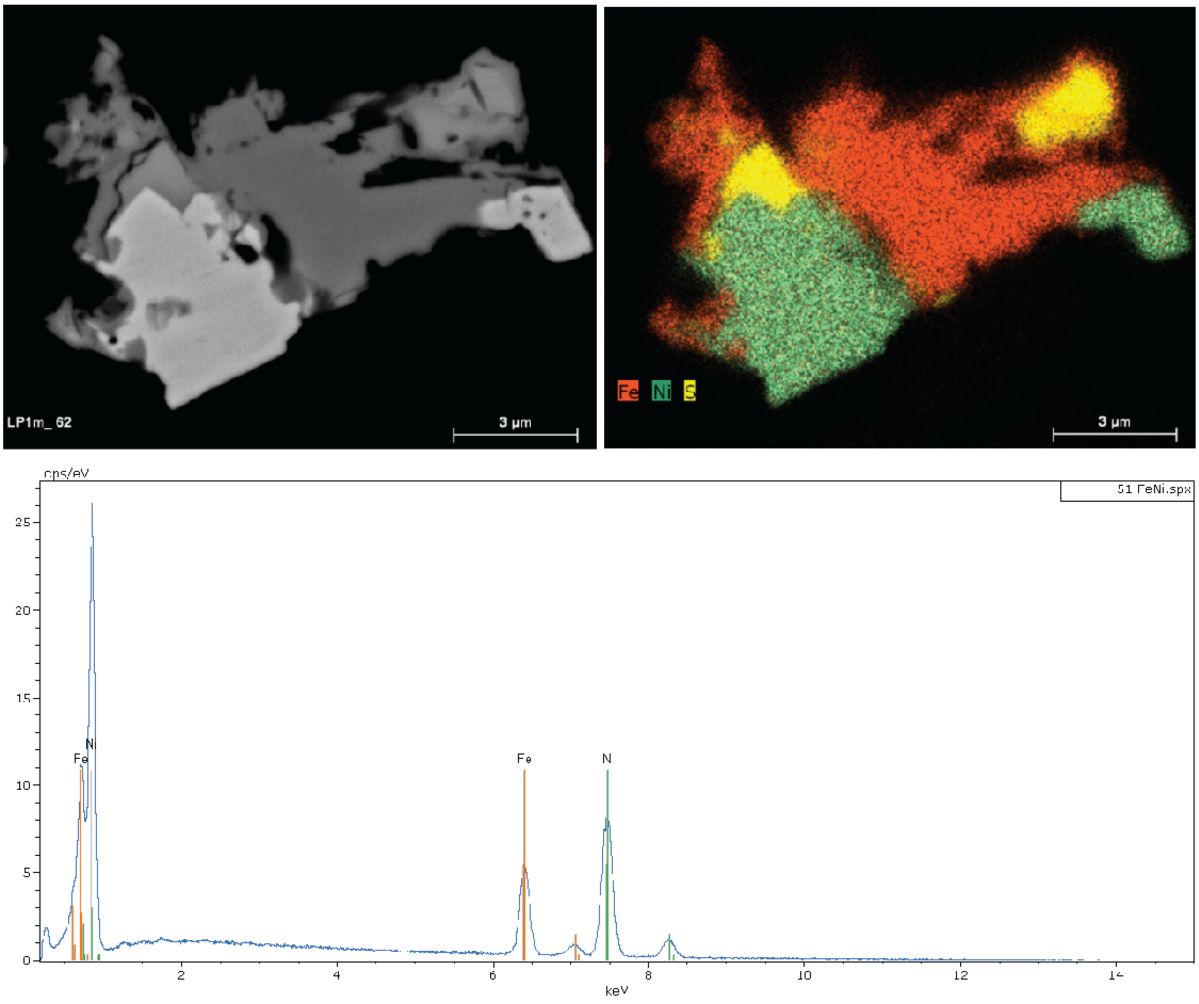

Supplementary Figure 9. Example of Fe-Ni alloy (green on the (EDX)-based mineralogical map to the right) in association with Fe-Ni-sulphide (yellow) and magnetite (red) in sample COR18-39f. The X-ray spectrum of awaruite is also shown. The microstructure suggests the early formation of awaruite, followed by the Fe-Ni-sulphide and magnetite.

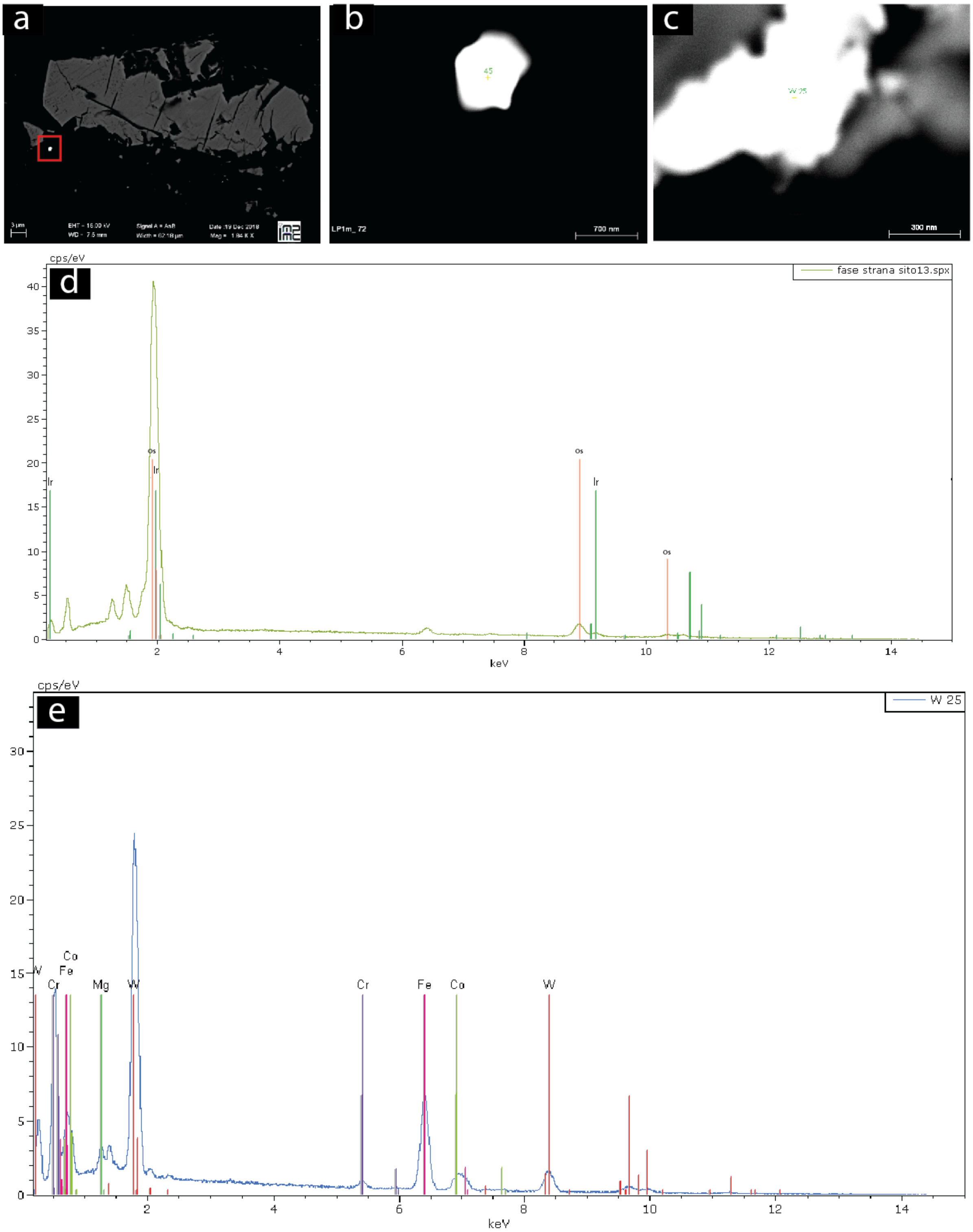

Supplementary Figure 10. a-c) Backscattered-electron images of Ir-Os (**a-b**) and W (**c**) alloys included in association with serpentine and magnetite. The X-Ray spectrum of the Ir-Os and W alloy particles is also shown in (**d**) and (**e**), respectively.

Supplementary Note 3

Thermodynamic modelling

High-pressure serpentinization was simulated with the Deep Earth Water (DEW) model[13] and the EQ3/EQ6 software[14] with a modified Berman thermodynamic database[15] (see also Supplementary Note 3 for additional information and discussion). Firstly we calculated with EQ3 the composition of a fluid in equilibrium with a serpentinite assemblage consisting of antigorite + magnetite + brucite + chlorite + olivine, which best represents the general mineralogical composition of serpentinites at blueschist-to-eclogite-facies conditions[16,17]. The fO_2 of the equilibrium was set at QFM (quartz-fayalite-magnetite buffer). The initial value of fluid fO_2 was chosen on the basis the predicted fO_2 conditions for a fluid equilibrated with the assemblage antigorite + magnetite + olivine + chlorite + brucite by Piccoli et al.[18]. In our oceanic serpentinite samples hematite was never observed, indicating that fO_2 conditions never exceeded the hematite-magnetite buffer. Nevertheless, it is worth noting that the chosen initial fluid fO_2 does not affect the final fO_2 condition at equilibrium after fluid-rock interaction. Choosing higher initial fO_2 condition will only lead to higher ΔfO_2 (Fig. 4c in the main text), thus our initial fO_2 conditions give more conservative results.

The molality of carbon in the fluid was set at values between 0.001 and 0.05, which encompass the values of carbon molality for in equilibrium with carbonate-undersaturated to carbonate saturated serpentinite at 400-500 °C and 1-2 GPa based on the EQ3 calculations. Modelled fluid component speciation in equilibrium with carbonate saturated serpentinites at 400 °C and 1 GPa and 500 °C and 2 GPa are presented in Table S2. EQ6 was then used to model the interaction between the EQ3 fluid and a harzburgite assemblage consisting of olivine, orthopyroxene, clinopyroxene, and spinel. The composition of the main solid solutions in the EQ3 and EQ6 calculations were set based on the mineral compositions analysed in the samples. Fluid/rock ratios from 1 to 10 were considered.

400 °C, 1 GPa c=0.02		500 °C, 2 GPa c=0.05	
	**Molal concentration**		**Molal concentration**
**$Mg(OH)_2(AQ)$**	3.74E-01	**$MgO(AQ)$**	1.82E+00
**$CO_2(AQ)$**	1.43E-02	**$CO_2(AQ)$**	2.06E-02
**$CH_4(AQ)$**	3.48E-03	**OH^-**	1.88E-02
**OH^-**	1.33E-03	**$Mg(SiO_2)HCO_3^+$**	1.88E-02
**HCO_3^-**	1.10E-03	**HCO_3^-**	5.20E-03
**$H_2(AQ)$**	1.07E-03	**$Fe(HCOO)^+$**	4.08E-03
**Cl^-**	9.92E-04	**$SiO_2(AQ)$**	2.07E-03
**Na^+**	9.88E-04	**Cl^-**	9.98E-04
**$H_2S(AQ)$**	7.27E-04	**Na^+**	9.59E-04
**$Ca(HCO_3)^+$**	5.38E-04	**$Mg(OH)^+$**	7.04E-04
**Ca^{++}**	3.73E-04	**$Ca(HCO_3)^+$**	6.22E-04
**$Fe(HCOO)^+$**	3.50E-04	**$H_2(AQ)$**	5.21E-04
**$Fe(H_3SiO_4)^+$**	3.07E-04	**$H_2S(AQ)$**	5.05E-04
**Mg^{++}**	3.03E-04	**HS^-**	4.94E-04
**$SiO_2(AQ)$**	2.75E-04	**$HCOO^-$**	4.55E-04
**HS^-**	2.73E-04	**$Fe(H_3SiO_4)^+$**	4.43E-04
**$Mg(SiO_2)HCO$**	1.22E-04	**$HSiO_3^-$**	1.90E-04
**$HCOO^-$**	6.30E-05	**$Ca(OH)^+$**	1.28E-04
**$Ca(OH)^+$**	4.14E-05	**Ca^{++}**	1.10E-04
**$Ca(H_3SiO_4)^+$**	1.72E-05	**Mg^{++}**	1.07E-04
**$Mg(HCO_3)^+$**	1.63E-05	**$CH_4(AQ)$**	7.78E-05
**$Ca(HCOO)^+$**	1.44E-05	**$Ca(H_3SiO_4)^+$**	7.02E-05
**$NaHCO_3(AQ)$**	1.07E-05	**CO_3^{--}**	5.20E-05
**$CaCO_3(AQ)$**	1.04E-05	**$Ca(HCOO)^+$**	3.77E-05
		**AlO_2^-**	3.74E-05
		**$NaHCO_3(AQ)$**	3.19E-05
		**$CaCO_3(AQ)$**	3.06E-05
		**$Mg(HCO_3)^+$**	1.80E-05

Supplementary Table 2. Fluid component speciation in equilibrium with carbonate-saturated serpentinites. Carbon molalities of 0.02 and 0.05 represent the threshold for carbonate saturations at 400 °C/1 GPa and 500 °C/2 GPa, respectively. Concentrations lower than 10^{-5} are not reported.

Complementary calculations were performed with the Perple_X software package[19], version 6.8.3. The following solution models were used: Olivine: O(HP); Orthopyroxene: Opx(HP); Clinopyroxene: Cpx(HP); Chlorite: Chl(HP); Spinel: Sp(HP); Garnet: Gt(HP)[20]; Antigorite: Atg(PN)[21]; Talc: T (talc + f-talc); Brucite: B (ideal); Tremolite: Tr (ideal); Antophyllite: Anth (ideal); Magnetite: MF (ideal); fluid: COH-fluid[22]; Wustiste: Wus. The pseudosection was built in order to reproduce a partially serpentinized (~50vol.% serpentine) harzburgite. The modelled system is (in moles) MgO = 1, SiO_2 = 0.7, Al_2O_3 = 0.01; CaO = 0.02, MnO = 0.001, FeO = 0.1 (spinel harzurgite MM2/11 in Rampone et al.[23]); 0.4 moles of H_2O were added to the system in order to reach about 50 vol.% serpentinization.

F/R=1								F/R=2		F/R=5		F/R=10	
**400 1GPa c=0.001**		**450 1GPa c=0.001**		**500 1GPa c=0.001**		**550 1GPa c=0.001**							
**Mt**	0.7	**Mt**	0.3	**Mt**	0.001	**Mt**	0.002	Not calculated		Not calculated		Not calculated	
**Atg**	29.2	**Atg**	46.9	**Atg**	34.0	**Atg**	9.7						
**Opx**	12.36	**Ol (Fo90)**	35.7	**Ol (Fo90)**	36.6	**Ol (Fo90)**	38.4						
**Ol (Fo90)**	46.9	**M-Ol (Fo64)**	7.9	**M-Ol (Fo82)**	19.5	**M-Ol (Fo91)**	39.6						
**M-Ol(Fo52)**	1.2	**Cpx**	5.8	**Cpx**	6.4	**Cpx**	8.7						
**Cpx**	5.9	**Chl**	3.4	**Chl**	3.5	**Chl**	3.7						
**Chl**	3.7	**Tot**	100	**Tot**	100	**Tot**	100						
**Tot**	100												
**400 1GPa c=0.02**		**450 1GPa c=0.02**		**500 1GPa c=0.02**		**550 1GPa c=0.02**		**400 1GPa c=0.02**		**400 1GPa c=0.02**		**400 1GPa c=0.02**	
**Mt**	1.1	**Mt**	1.5	**Mt**	0.001	**Mt**	0.002	**Mt**	1.4	**Mt**	4.2	**Mt**	4.2
**Atg**	50.8	**Atg**	51.9	**Atg**	34.0	**Atg**	9.7	**Atg**	51.2	**Atg**	88.7	**Brc**	1.1
**Tr**	3.2	**Ol (Fo90)**	35.0	**Ol (Fo90)**	36.6	**Ol (Fo90)**	38.4	**Ol (Fo90)**	37.3	**Brc**	1.2	**Atg**	88.3
**Carb**	0.01	**M-Ol (Fo61)**	2.5	**M-Ol (Fo82)**	19.5	**M-Ol (Fo91)**	39.6	**M-Ol(Fo52)**	1.8	**Ol (Fo90)**	—	**Ol (Fo90)**	—
**Ol (Fo90)**	35.39	**Cpx**	5.8	**Cpx**	6.4	**Cpx**	8.6	**Cpx**	4.9	**M-Ol**	—	**M-Ol**	—
**M-Ol(Fo33)**	1.8	**Chl**	3.4	**Chl**	3.5	**Chl**	3.7	**Chl**	3.4	**Cpx**	2.9	**Cpx**	3.5
**Cpx**	4.3	**Tot**	100	**Tot**	100	**Tot**	100.0	**Tot**	100	**Chl**	3.0	**Chl**	3.0
**Chl**	3.4									**Tot**	100	**Tot**	100
**Tot**	100												

F/R=1								F/R=5		F/R=10	
**400 2GPa c=0.001**		**450 2GPa c=0.001**		**500 2GPa c=0.001**		**550 2GPa c=0.001**		**500 2GPa c=0.001**		**500 2GPa c=0.001**	
**Mt**	0.1	**Mt**	0.1	**Mt**	0.018	**Mt**	0.001	**Mt**	0.0004	**Mt**	0.001
**Atg**	52.6	**Atg**	46.7	**Atg**	39.6	Atg	19.4	**Atg**	18.7	**Atg**	19.42
**Pyr**	0.0001	**Ol (Fo90)**	35.6	**Ol (Fo90)**	36.6	**Ol (Fo90)**	38.0	**Ol (Fo90)**	52.3	**Ol (Fo90)**	36.9
**Ol (Fo90)**	35.7	**M-Ol (Fo65)**	8.8	**M-Ol (Fo80)**	17.6	**M-Ol (Fo89)**	35.2	**M-Ol (Fo89)**	25.0	**M-Ol (Fo91)**	39.5
**M-Ol (Fo47)**	6.0	**Cpx**	5.3	**Cpx**	5.8	**Cpx**	6.9	**Cpx**	1.4	**Cpx**	0.6
**Cpx**	5.1	**Chl**	3.4	**Chl**	0.4	**Chl**	0.4	**Chl**	2.6	**Chl**	3.5
**Chl**	0.3	**Tot**	100	**Tot**	100	**Tot**	100	**Tot**	100	**Tot**	100
**Tot**	100										
**400 2GPa c=0.02**		**450 2GPa c=0.02**		**500 2GPa c=0.02**		**550 2GPa c=0.02**		**500 2GPa c=0.02**		**500 2GPa c=0.02**	
**Mt**	0.9	**Mt**	1.4	**Mt**	0.100	**Atg**	24.0	**Mt**	—	**Mt**	—
**Atg**	54.6	**Atg**	54.4	**Atg**	40.4	**Ol (Fo90)**	47.2	**Atg**	26.6	**Atg**	21.3
**Ol (Fo90)**	35.3	**Ol (Fo90)**	35.1	**Ol (Fo90)**	36.5	**M-Ol (89)**	43.7	**Ol (Fo90)**	36.0	**Ol (Fo90)**	36.4
**M-Ol (Fo46)**	3.75	**M-Ol (Fo64)**	3.45	**M-Ol (Fo80)**	16.9	**Cpx**	8.7	**M-Ol (Fo89)**	32.8	**M-Ol (Fo90)**	38.8
**Cpx**	5.1	**Cpx**	5.2	**Cpx**	5.7	**Chl**	0.4	**Cpx**	1.2	**Cpx**	0.0
**Chl**	0.3	**Chl**	0.3	**Chl**	0.3	**Tot**	100	**Chl**	3.4	**Chl**	3.5
**Carb**	0.1	**Tot**	100	**Tot**	100			**Tot**	100	**Tot**	100
**Tot**	100										
				**500 2GPa c=0.05**							
				**Mt**	0.240						
				**Atg**	42.0						
				**Ol (Fo90)**	36.5						
				**M-Ol (Fo80)**	15.8						
				**Cpx**	5.6						
				**Chl**	0.3						
				**Tot**	100						

SupplementaryTable 3. Mineral assemblages at reaction completion as predicted by the DEW model calculations in vol.%.

Effect of C content on serpentinization

Each P-T run was calculated for two different initial C concentrations in the serpentinizing fluid corresponding to 0.001 (strongly undersaturated with respect to carbonate minerals), 0.02 and 0.05 molal —for reference, 0.02 molal corresponds to an X_{CO2} = 0.0004—. The latter two concentrations roughly correspond to the saturation of carbonate minerals in the rock in during the fluid/rock interaction at 1 and 2 GPa, respectively, as predicted for carbon sequestration in the mantle wedge[24-26].

The calculations show that higher carbon concentrations in the infiltrating fluid enhances the production of antigorite and magnetite (Table S3).

Effect of P, T, and fluid/rock ratio

Our calculations show that serpentinization is effective to T of at least 550 °C at 1 GPa, and < 550 °C at 2GPa. Fluid/rock rations higher than those considered in this study may enlarge the pressure-temperature range of high-pressure serpentinization. Fluid/rock ratios > 1 generally result in either higher or lower degrees of serpentinization at 1 GPa and 2GPa, respectively (Table S3). In either case, higher fluid/rock ratios results in lower H_2 and CH_4 concentrations at reaction completion (Table S4).

	F/R=1				F/R=2	F/R=5	F/R=10
	**400 1GPa c=0.001**	**450 1GPa c=0.001**	**500 1GPa c=0.001**	**550 1GPa c=0.001**			
**CH_4(AQ)**	1.02E-03	1.01E-03	2.18E-05	9.93E-06			
**CO_2(AQ)**	3.11E-09	1.99E-05	9.22E-04	9.54E-04			
**H_2(AQ)**	3.65E-02	1.58E-02	7.93E-03	1.98E-02			
**H_2S(AQ)**	4.31E-04	6.83E-04	8.30E-04	8.97E-04	Not calculated	Not calculated	Not calculated
**SO_4^-**	4.11E-15	3.47E-12	1.08E-09	3.56E-10			
**$CaCO_3$(AQ**	—	4.99E-08	3.65E-07	2.61E-08			
**HCO_3^-**	8.72E-10	1.16E-06	1.36E-05	3.81E-06			
	**400 1GPa c=0.02 N=10^{-6}**	**450 1GPa c=0.02**	**500 1GPa c=0.02**	**550 1GPa c=0.02**	**400 1GPa c=0.02**	**400 1GPa c=0.02**	**400 1GPa c=0.02**
**CH_4(AQ)**	1.93E-02	2.00E-02	4.35E-04		1.02E-03	1.02E-03	1.01E-03
**CO_2(AQ)**	5.36E-04	5.93E-04	1.85E-02	1.91E-02	2.60E-09	7.53E-10	1.59E-08
**H_2(AQ)**	3.74E-03	2.77E-02	7.91E-03	1.99E-02	3.82E-02	5.21E-02	2.42E-02
**H_2S(AQ)**	4.43E-04	6.85E-04	8.17E-04	8.95E-04	4.26E-04	2.75E-04	2.72E-04
**SO_4^-**	3.71E-11	5.29E-12	1.27E-09	3.71E-10	3.52E-15	2.68E-15	5.68E-14
**$CaCO_3$(AQ**	7.57E-07	1.10E-06	7.31E-06	5.22E-07	—	1.82E-10	3.87E-09
**HCO_3^-**	2.95E-05	3.49E-05	2.96E-04	7.83E-05	7.41E-10	1.16E-09	8.84E-09
**NH_3**	9.67E-07						
**NH_4^+**	7.24E-08						
**N_2**	2.57E-12						
**NO_3^-**	1.17E-43						
	**400 2GPa c=0.001**	**450 2GPa c=0.001**	**500 2GPa c=0.001**	**550 2GPa c=0.001**		**500 2GPa c=0.001***	**500 2GPa c=0.001**
**CH_4(AQ)**	1.04E-03	1.03E-03	3.58E-05	1.51E-06		1.51E-06	1.50E-06
**CO_2(AQ)**	4.84E-10	3.37E-07	3.58E-05	4.42E-04		2.35E-04	2.28E-04
**H_2(AQ)**	4.20E-03	3.85E-03	1.32E-03	1.81E-03		5.94E-04	5.99E-04
**H_2S(AQ)**	5.59E-05	2.75E-04	5.26E-04	3.77E-04	Not calculated	4.49E-04	4.36E-04
**SO_4^-**	3.69E-14	8.43E-12	2.13E-08	5.53E-08		7.55E-07	7.89E-07
**$CaCO_3$(AQ**	1.06E-09	1.29E-07	1.15E-05	2.10E-06		2.44E-05	2.70E-05
**HCO_3^-**	3.44E-09	4.39E-07	5.65E-05	2.27E-05		7.44E-05	7.57E-05
	**400 2GPa c=0.02**	**450 2GPa c=0.02**	**500 2GPa c=0.02**	**550 2GPa c=0.02**		**500 2GPa c=0.02 N=10-**	**500 2GPa c=0.001**
**CH_4(AQ)**	1.23E-02	2.07E-02	1.02E-03	7.49E-05		6.75E-05	7.40E-05
**CO_2(AQ)**	1.06E-08	7.73E-06	5.07E-03	9.78E-03		5.05E-03	5.24E-03
**H_2(AQ)**	3.59E-03	3.73E-03	1.41E-03	2.22E-03		7.15E-04	7.24E-04
**H_2S(AQ)**	6.31E-05	2.85E-04	4.73E-04	3.49E-04		4.06E-04	4.19E-04
**SO_4^-**	6.46E-14	9.13E-12	3.85E-07	8.98E-08		4.79E-07	4.14E-07
**$CaCO_3$(AQ**	1.87E-08	2.62E-06	2.33E-04	4.09E-08	Not calculated	5.17E-04	4.69E-04
**HCO_3^-**	6.92E-08	9.66E-06	1.57E-03	6.61E-04		1.91E-03	1.87E-03
**NH_3**						8.79E-07	
**NH_4^+**						1.25E-07	
**N_2**						4.57E-10	
**NO_3^-**						4.00E-35	
			**500 2GPa c=0.05**			**500 2GPa c=0.001**	**500 2GPa c=0.001**
**CH_4(AQ)**			2.19E-03				
**CO_2(AQ)**			1.40E-02				
**H_2(AQ)**			1.32E-03				
**H_2S(AQ)**	Not calculated		4.01E-04	Not calculated	Not calculated	Not calculated	Not calculated
**SO_4^-**			4.75E-08				
**$CaCO_3$(AQ**			7.06E-04				
**HCO_3^-**			5.67E-03				

SupplementaryTable 4. Modelled fluid component speciation for selected fluid species. All values in mol/kg of fluid. Nitrogen speciation was calculated for two pressure-temperature conditions (400 °C/1 GPa and 500 °C/2 GPa). * refers to antigorite-free runs.

Similarly, it was proposed that for low-temperature (and pressure) serpentinization the H_2 concentration decreases with increasing degree of serpentinization (> 75%)[27]. The concentrations of H_2 and CH_4 are therefore not only related to antigorite formation at the expense of olivine (Table S4). For H_2 and CH_4 production proposed in the text (see below), we considered only pressure and temperature conditions characterized by antigorite-bearing assemblages. Brucite is generally predicted for fluid/rock ratios > 5, and not observed together with metamorphic olivine (Table S3). The Mg# of metamorphic olivine increases with increasing temperature (Table S3).

HCO_3^- and aqueous $CaCO_3$ increase relative to CH_4 at 2 GPa compared to 1 GPa. CO_2 increases with increasing temperature and reaches concentrations higher than CH_4 at T > 460 °C - 480 °C, which is the temperature expected at the blueschist-eclogite transition[1]. H_2S is the dominant S-bearing species and increases with temperature and decreases with P (Table S4).

H_2 and CH_4 production rates

We considered the amount of bound water released annually from the slab to 100km depth and interacting with the overlying mantle wedge from van Keken et al. [28] ($3.3 \cdot 10^{12}$ kg/year). We assumed fluid/rock ratios equal to 1, which at 400°C and 1 GPa correspond to a serpentinization degree of 50 vol.%. This value represents the upper bound for the degree of mantle wedge serpentinization proposed by Hyndman and Peacock[29] (20-50 vol.%). Assuming a fluid/rock ratio equal to 1 means that each kg of H_2O released from the slab interact with 1 kg or peridotite and resulting in partial serpentinization. Thereby, considering a density of 3.3 g/cm^3 for the mantle wedge, the annual H_2O flux from the slab results in partial serpentinization of about 1 km^3 of mantle wedge, which appears a realistic value at the global scale. We used the H_2 and CH_4 concentrations at reaction completion resulting from the interaction of 1 kg of peridotite with 1 kg of fluid, as modelled via EQ3/EQ6. This way, each kg of H_2O released from the slab

and interacting with the mantle wedge corresponds to a given amount of produced H_2. We considered the smallest and biggest H_2 concentrations reported in Fig. 4d, which correspond to 500 °C and 2GPa and 400 °C and 1GPa, respectively. The calculation provides a lowermost and uppermost bound of $9 \cdot 10^{-3}$ and 0.3 Mt/year for H_2, respectively. For CH_4, we considered different C concentrations in the serpentinizing fluid, 0.001, 0.02, and 0.05 molal (see above for details).

This model includes the following assumptions and potential biases.

- The amount of slab-derived H_2O interacting with the mantle wedge from van Keken et al. [28] refers to the total amount of bound water released to 100 km depth. In our case, we considered antigorite serpentinization happening at depth > 35-40 km, and therefore H_2O release from 35-40 to 100 km depth, which may result in overestimation of H_2 and CH_4 production. Nevertheless, the amount of bound H_2O release at depth < 40 km is minimal, and it can be assumed that most of the H_2O release to 100 km depth happens in the 40 to 100 km range (with the exception of the warmest thermal regimes).
- The model assumes uniform fluid-rock interaction. Channelized fluid pathways may result in H_2O saturation and complete serpentinization of localized domains of the mantle wedge with no further fluid-rock interaction.
- The model for antigorite currently included in the DEW package does not include Fe, which may result in significant overestimation of magnetite and related H_2 production. This effect can be in part reduced by the low Fe concentration in antigorite (2-3 wt.%, Table S1). Moreover, blueschist and eclogite-facies antigorite in Alpine serpentinites generally has a quite high Fe^{3+}/Fe_{Tot} ratio (0.3-0.4), which would imply H_2 generation. Nevertheless, given the potential uncertainty on the magnetite and H_2 estimates predicted by the DEW/EQ3-6 models, we performed complementary pseudosection calculations with the Perple_X package (Supplementary Figure 11). The results show

that the predicted magnetite amounts for a ~ 50 vol.% serpentinized peridotite at 400 °C and 1 GPa (~ 2.8 vol.%) are similar or even higher to those obtained via the DEW/EQ3-6 models (~ 2 vol.%). The absence of Fe in antigorite also affects its stability to high-temperature conditions. In particular, our modelling results underestimate the potential for high-pressure serpentinization to extend to T > 500 0°C, and therefore the conditions suitable for H_2 production.

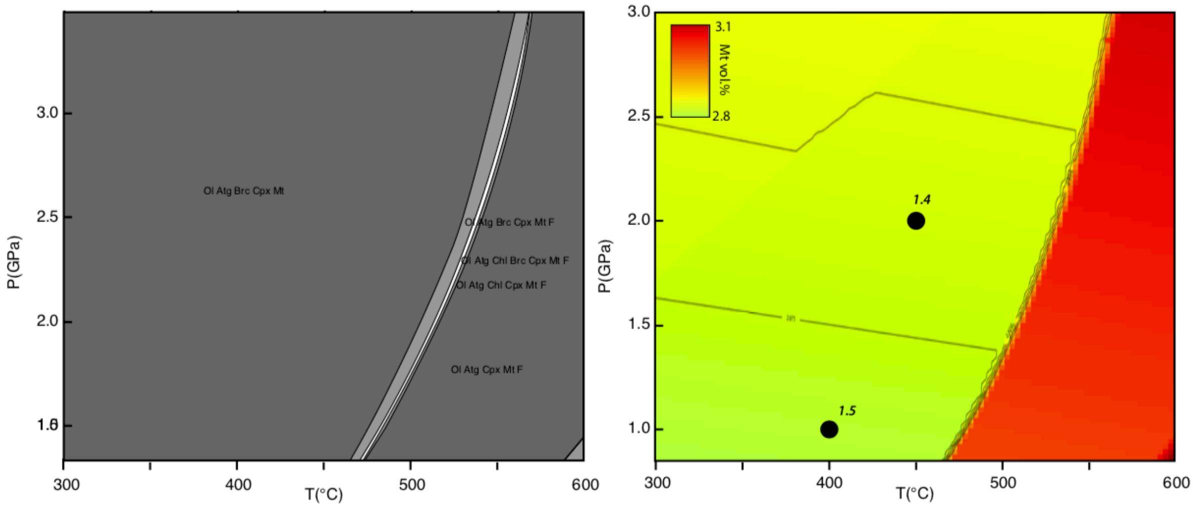

Supplementary Figure 11. Pseudosection and calculated magnetite vol.% obtained with Perple_X for a 50% serpentinization of a harzburgite. The black dot and related numbers (in italics) refer to the magnetite vol.% obtained with the DEW model for the same conditions (Supplementary Table 3) and roughly equivalent serpentinization degree.

- The values of H_2 and CH_4 produced per kg of H_2O correspond to the concentration of these species in the last reaction step of the EQ6 runs considering and not the cumulative H_2-CH_4 production through equilibration. The cumulative amounts of H_2 and CH_4 could greatly increase the estimated fluxes.

- The global H_2 production model was constructed for fluid/rock rations equal to 1, with serpentinization degrees not exceeding ~ 50%. Greater fluid/rock ratios and complete serpentinization result in higher H_2 and CH_4 concentrations.
- The global H_2 production model refers uniquely to mantle wedge serpentinization. Slab serpentinization, as observed in the selected case studies, can produce significant amounts of H_2 interacting with carbonate-rock rocks in the slab and producing potentially high concentrations of CH_4[30]. Additional H_2 and CH_4 sources can exist in the subducted mafic crust[31], and at antigorite + brucite and chlorite-harzburgite dehydration[18].

Overall, the above considerations make our model conservative.

Supplementary Note 4

Pressure and temperature conditions of HP serpentinization in modern and ancient subduction zones

Supplementary Figure 12 shows the peak metamorphic conditions of subduction-related rocks from the Neoarchean to the Phanerozoic[32] plotted onto the stability field of antigorite[33]. The predicted P-T path of the slab surface in active subduction zones[34] is also shown (model D80). Cold subduction zones, characteristic of the Phanerozoic, are characterized by serpentinite (and therefore serpentinization) stability extending to about 100 km depth, but potentially low degrees of serpentinization at least in the mantle wedge[35]. These conditions are favourable for high H_2-CH_4 concentrations in the fluid. Slab serpentinization in cold subduction zones such as the Alpine subduction can, however, be pervasive, as recorded by the studied samples.

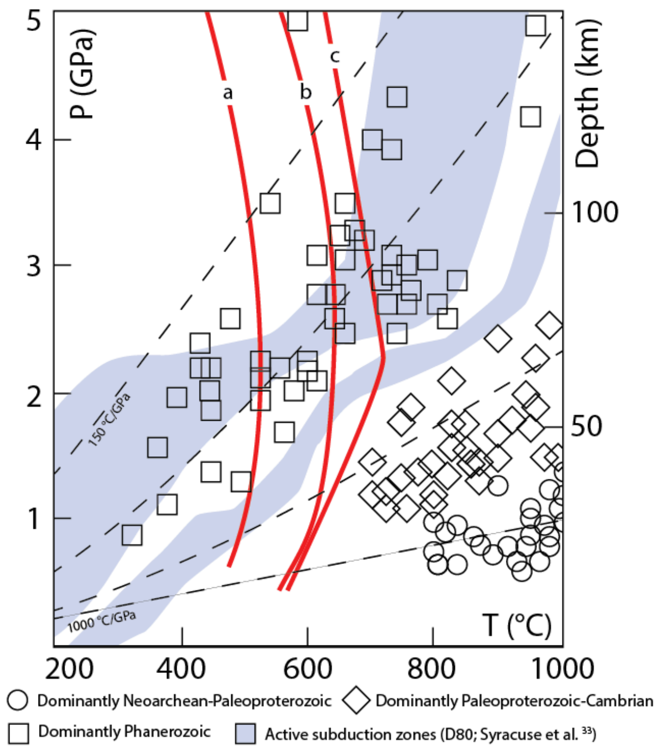

Supplementary Figure 12. Range pressure (P) and temperature (T) conditions suitable for subduction serpentinization in modern and ancient subduction zones. See text for details and references. Curves a, b, and c represent selected dehydration reactions of serpentine minerals from Guillot et al.[33] and references therein. Pressure-temperature paths for active subduction zones are from Syracuse et al. [34] (Model D80).

In warm subduction zones, which were dominant in the Neoarchean-Paleoproterozoic but are also present in active subduction zones, the stability field of serpentine/serpentinization is restricted to much shallower depths (< 35 km) compared to cold subduction zones. Serpentinization of the mantle wedge in warm subduction zones is predicted to more pronounced compared to cold subduction zones[35]. These conditions are suitable for high integrated H_2 and CH_4 fluxes, but potentially lower H_2-CH_4 concentrations in the fluid.

Supplementary Note 5

Fluid inclusion analysis

The fluid inclusions were analysed by MicroRaman spectroscopy (cf. Methods). The analysed fluid inclusions show a variety of fluid species, even within a single inclusion trail. Methane is ubiquitous in all samples (Supplementary Figure 13). The presence of H_2 was identified in all the three selected localities, even though it was not systematically detected in all analysed fluid inclusions. Water/vapor was not detected, even though thin H_2O films may be present in the fluid inclusions[36,37]. The initial presence of H_2O is inferred based on the identification of hydrous daughter minerals such as serpentine (lizardite) and brucite, which were identified based on the characteristic O-H stretching bands at 3683-3698 and 3645-3652 cm^{-1}, respectively (Supplementary Figure 13). The presence of lizardite instead of antigorite suggests a relatively late post-entrapment hydration of the inclusion walls (T < 380 °C). The initial presence of reduced fluid species during the entrapment is indicated by the non-systematic presence of daughter minerals in the inclusions. Moreover, no magnetite was detected, suggesting that the H_2 identified in the inclusion was most likely already present in the original fluid. Ethane (C_2H_6) was detected in one sample from Lanzo (Supplementary Figure 13). The analysis was made on a thin section and was affected by epoxy contamination. Nevertheless, a detailed comparison between the spectra of the fluid inclusion and the epoxy from the same samples indicate the actual presence of the C_2H_6 characteristic band at 2954 $cm^{-}1$ (Supplementary Figure 13). Nitrogen was detected in fluid inclusions from Cima di Gratera and San Petrone. In the Cima di Gratera rocks, only N_2 was detected. The effect of atmospheric N_2 contamination was checked with equivalent analytical protocol on the host olivine and appeared negligible. Ammonia (NH_3) was systematically detected in the samples from San Petrone (Supplementary Figure 13). The presence of N_2 was found to be more scattered and potentially resulting from partial reequilibration of NH_3 in the inclusions. Two unidentified compounds

were also detected at 3422 cm^{-1} and 3000 cm^{-1}, which fall within the N-H stretching region. The presence of reduced C particles in the Lanzo samples is suggested by the appearance of the characteristic C-C stretching bands (Supplementary Figure 13). The effect of epoxy contamination was also tested in this case and did not show bands overlapping with the inferred reduced C particles.

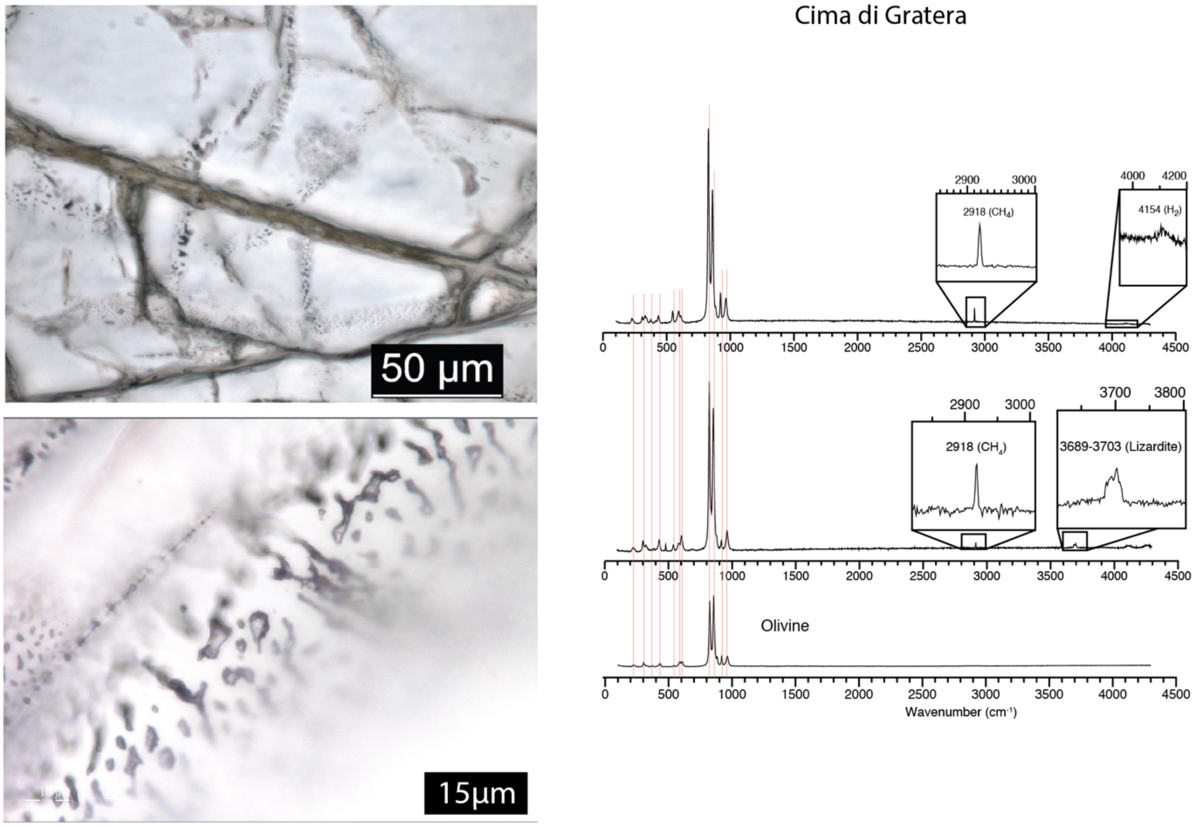

Supplementary Figure 13. Selected MicroRaman spectra of fluid inclusions from the three selected case studies. See text for details.

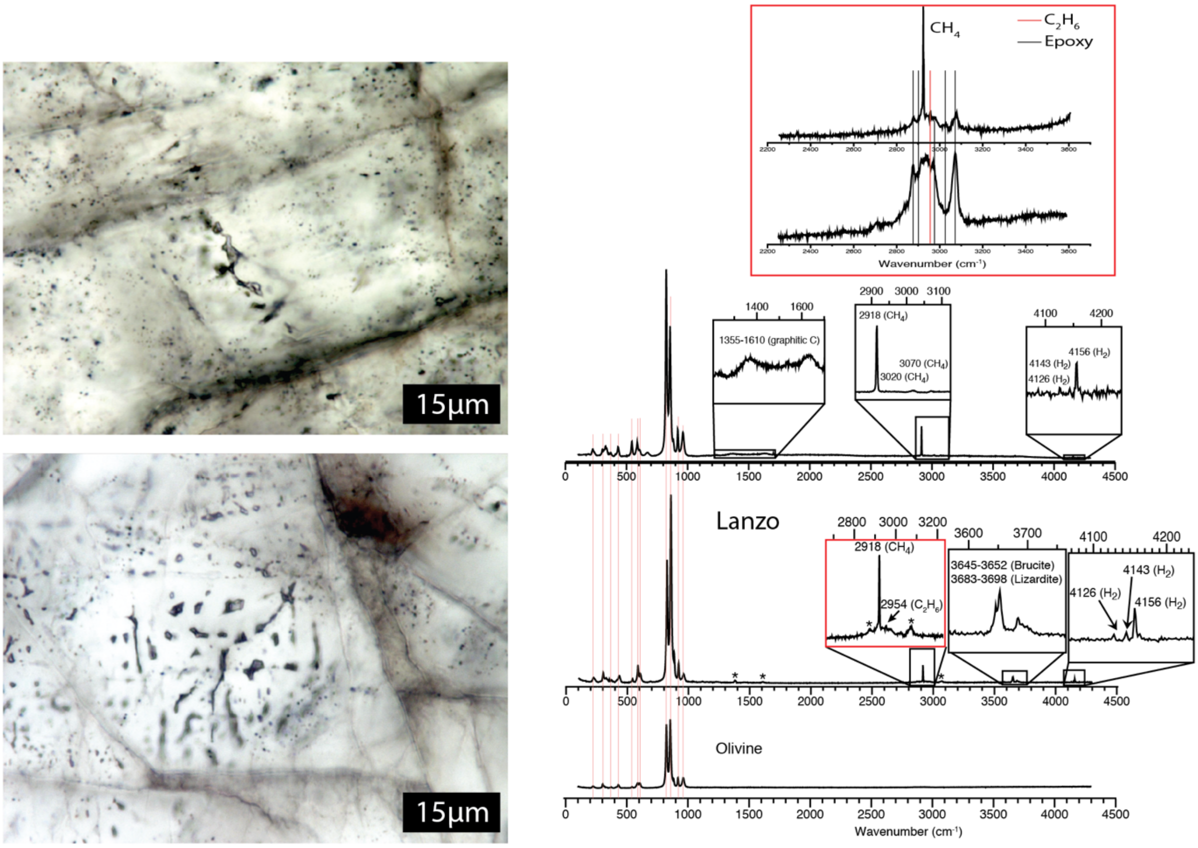

Supplementary Figure 13. Continued.

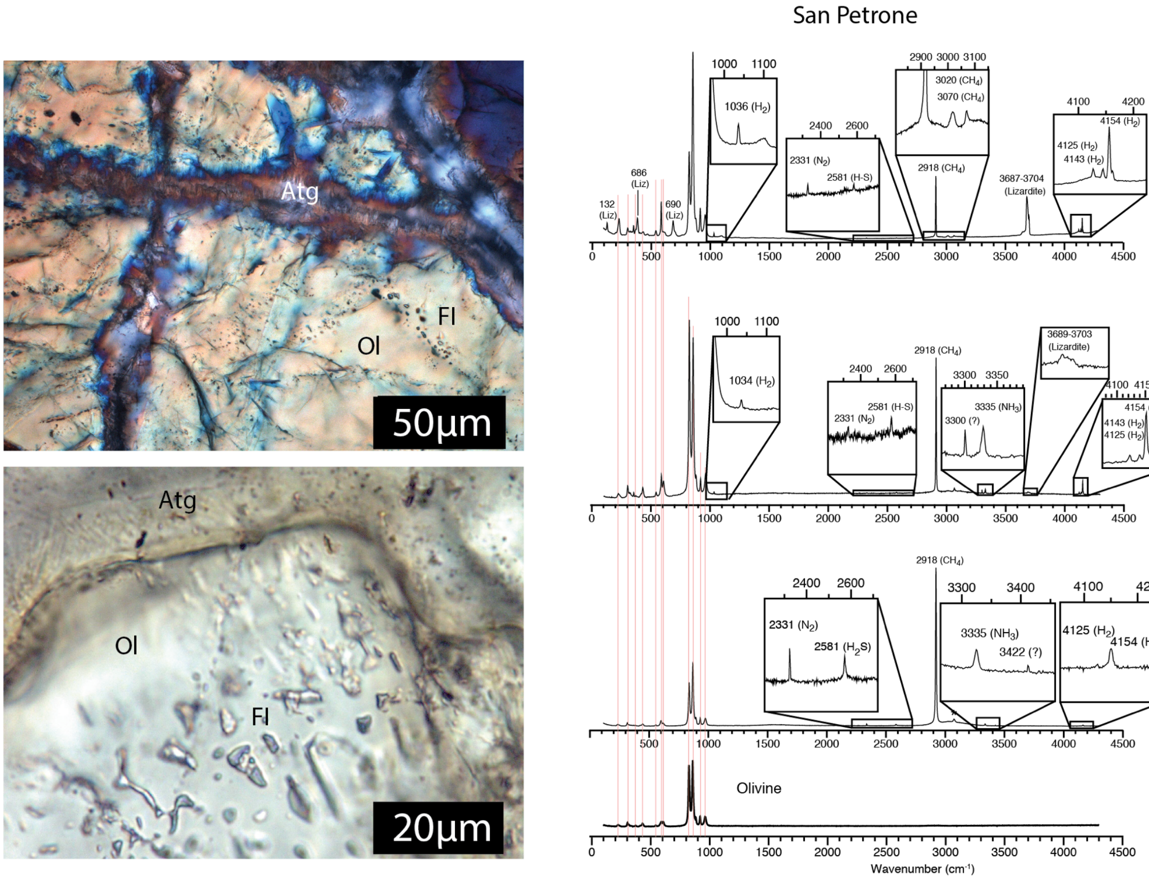

Supplementary Figure 13. Continued.

Supplementary References

1. Vitale Brovarone, A., Picatto, M., Beyssac, O., Lagabrielle, Y. & Castelli, D. The blueschist–eclogite transition in the Alpine chain: P–T paths and the role of slow-spreading extensional structures in the evolution of HP–LT mountain belts. *Tectonophysics* **615-616,** 96–121 (2014).
2. Vitale Brovarone, A. *et al.* Stacking and metamorphism of continuous segments of subducted lithosphere in a high-pressure wedge: The example of Alpine Corsica (France). *Earth Science Reviews* **116,** 35–56 (2013).
3. Vitale Brovarone, A. *et al.* Inherited Ocean – Continent Transition zones in deeply subducted terranes : Insights from Alpine Corsica. *Lithos* **124,** 273–290 (2011).
4. Beltrando, M. *et al.* Recognizing remnants of magma-poor rifted margins in high-pressure orogenic belts: The Alpine case study. *Earth Science Reviews* **131,** 88–115 (2014).
5. Deseta, N., Andersen, T. B. & Ashwal, L. D. A weakening mechanism for intermediate-depth seismicity? Detailed petrographic and microtextural observations from blueschist facies pseudotachylytes, Cape Corse, Corsica. *Tectonophysics* 1–12 (2013). doi:10.1016/j.tecto.2013.11.007

6. Deseta, N., Ashwal, L. D. & Andersen, T. B. Initiating intermediate-depth earthquakes: Insights from a HP–LT ophiolite from Corsica. *Lithos* **206-207,** 127–146 (2014).
7. Magott, R., Fabbri, O. & Fournier, M. Polyphase ductile/brittle deformation along a major tectonic boundary in an ophiolitic nappe, Alpine Corsica: Insights on subduction zone intermediate-depth asperities. *Journal of Structural Geology* **94,** 240–257 (2017).
8. Magott, R., Fabbri, O. & Fournier, M. Subduction zone intermediate-depth seismicity: Insights from the structural analysis of Alpine high-pressure ophiolite-hosted pseudotachylyte (Corsica, France). *Journal of Structural Geology* **87,** 95–114 (2016).
9. Piccardo, G. B., Ranalli, G. & Guarnieri, L. Seismogenic Shear Zones in the Lithospheric Mantle: Ultramafic Pseudotachylytes in the Lanzo Peridotite (Western Alps, NW Italy). *Journal of Petrology* **51,** 81–100 (2010).
10. Scambelluri, M. *et al.* Fossil intermediate-depth earthquakes in subducting slabs linked to differential stress release. *Nature Publishing Group* 1–8 (2017). doi:10.1038/s41561-017-0010-7
11. Andersen, T. B. & Austrheim, H. Fossil earthquakes recorded by pseudotachylytes in mantle peridotite from the Alpine subduction complex of Corsica. *Earth and Planetary Science Letters* **242,** 58–72 (2006).
12. Nakatani, T. & Nakamura, M. Experimental constraints on the serpentinization rate of fore-arc peridotites: Implications for the upwelling condition of the slab-derived fluid. *Geochemistry, Geophysics, Geosystems* **17,** 3393–3419 (2016).
13. Sverjensky, D. A., Harrison, B. & Azzolini, D. Water in the deep Earth: The dielectric constant and the solubilities of quartz and corundumto 60 kb and 1200 °C. *Geochimica et Cosmochimica Acta* **129,** 125–145 (2014).
14. Wolery, T. J. & Jerek, R. L. *Software User's Manual EQ3/6 (version 8.0). Sandia National Laboratories, Albuquerque, New Mexico* (2003).
15. Berman, R. G. Internally-Consistent Thermodynamic Data for Minerals in the System Na_2O-K_2O-CaO-MgO-FeO-Fe_2O_3-Al_2O3-SiO_2-TiO_2-H_2O-CO_2. *Journal of Petrology* **29,** 445–522 (1988).
16. Debret, B., Nicollet, C., Andréani, M., Schwartz, S. & Godard, M. Three steps of serpentinization in an eclogitized oceanic serpentinization front (Lanzo Massif - Western Alps). *Journal of metamorphic Geology* **31,** 165–186 (2012).
17. Schwartz, S. *et al.* Pressure–temperature estimates of the lizardite/antigorite transition in high pressure serpentinites. *Lithos* **178,** 197–210 (2013).
18. Piccoli, F. *et al.* Subducting serpentinites release reduced, not oxidized, aqueous fluids. *Scientific Reports* **9,** 1–7 (2019).
19. Connolly, J. A. D. Computation of phase equilibria by linear programming: a tool for geodynamic modeling and its application to subduction zone decarbonation. *Earth and Planetary Science Letters* **236,** 524–541 (2005).
20. Holland, T. J. B. & Powell, R. An internally consistent Interest., thermodynamic data set for phases of petrological interest. *Journal of metamorphic Geology* **16,** 309–343 (1998).
21. Padrón-Navarta, J. A. *et al.* Tschermak's substitution in antigorite and consequences for phase relations and water liberation in high-grade serpentinites. *Lithos* **178,** 186–196 (2013).
22. Connolly, J. A. D. & Galvez, M. E. Electrolytic fluid speciation by Gibbs energy minimization and implications for subduction zone mass transfer. *Earth and Planetary Science Letters* **501,** 90–102 (2018).

23. Rampone, E., Piccardo, G. B. & Hofmann, A. W. Multi-stage melt–rock interaction in the Mt. Maggiore (Corsica, France) ophiolitic peridotites: microstructural and geochemical evidence. *Contributions to Mineralogy and Petrology* **156,** 453–475 (2008).
24. Kelemen, P. B. & Manning, C. E. Reevaluating carbon fluxes in subduction zones, what goes down, mostly comes up. *PNAS* **112,** E3997–E4006 (2015).
25. Piccoli, F. *et al.* Carbonation by fluid–rock interactions at high-pressure conditions: Implications for carbon cycling in subduction zones. *Earth and Planetary Science Letters* **445,** 146–159 (2016).
26. Scambelluri, M. *et al.* Earth and Planetary Science Letters. *Earth and Planetary Science Letters* **441,** 155–166 (2016).
27. Klein, F. *et al.* Magnetite in seafloor serpentinite--Some like it hot. *Geology* **42,** 135–138 (2014).
28. van Keken, P. E., Hacker, B. R., Syracuse, E. M. & Abers, G. A. Subduction factory: 4. Depth-dependent flux of H 2O from subducting slabs worldwide. *Journal of Geophysical Research* **116,** B01401 (2011).
29. Hyndman, R. D. & Peacock, S. M. Serpentinization of the forearc mantle. *Earth and Planetary Science Letters* **212,** 417–432 (2003).
30. Vitale Brovarone, A. *et al.* Massive production of abiotic methane during subduction evidenced in metamorphosed ophicarbonates from the Italian Alps. *Nature Communications* **8,** 14134–13 (2017).
31. Tao, R. *et al.* Formation of abiotic hydrocarbon from reduction of carbonate in subduction zones: Constraints from petrological observation and experimental simulation. *Geochimica et Cosmochimica Acta* **239,** 390–408 (2018).
32. Brown, M. in *Special Paper 440: When Did Plate Tectonics Begin on Planet Earth?* **440,** 97–128 (Geological Society of America, 2008).
33. Guillot, S., Schwartz, S., Reynard, B., Agard, P. & Prigent, C. Tectonic significance of serpentinites. *Tectonophysics* **646,** 1–19 (2015).
34. Syracuse, E. M., van Keken, P. E. & Abers, G. A. The global range of subduction zone thermal models. *Physics of the Earth and Planetary Interiors* **183,** 73–90 (2010).
35. Abers, G. A., van Keken, P. E. & Hacker, B. R. The cold and relatively dry nature of mantle forearcs in subduction zones. *Nature Publishing Group* **10,** 333–337 (2017).
36. Berkesi, M. *et al.* Detection of small amounts of H_2O in CO_2-rich fluid inclusions using Raman spectroscopy. *J. Raman Spectrosc.* **40,** 1461–1463 (2009).
37. Lamadrid, H. M., Lamb, W. M., Santosh, M. & Bodnar, R. J. Raman spectroscopic characterization of H_2O in CO_2-rich fluid inclusions in granulite facies metamorphic rocks. *Gondwana Research* **26,** 301–310 (2014).
